# Trends in prevalence of fractures among adults in the United States, 1999–2020: a population-based study

**DOI:** 10.1097/JS9.0000000000000883

**Published:** 2023-11-03

**Authors:** Bin Xu, Maja R. Radojčić, David B. Anderson, Baoyi Shi, Liang Yao, Yujie Chen, Shiqing Feng, Jae Hyup Lee, Lingxiao Chen

**Affiliations:** a The Second Hospital of Shandong University; b Department of Orthopaedics, Qilu Hospital of Shandong University, Shandong University Centre for Orthopaedics, Advanced Medical Research Institute; c Department of Biostatistics, School of Public Health, Cheeloo College of Medicine, Shandong University, Jinan, Shandong, People's Republic of China; d Faculty of Medicine and Health, The University of Sydney, Sydney School of Health Sciences; e Sydney Musculoskeletal Health, Patyegarang Precinct; f Sydney Musculoskeletal Health, School of Health Sciences, Faculty of Medicine and Health, The University of Sydney, Sydney, New South Wales, Australia; g Program in Child Health Evaluative Sciences, The Hospital for Sick Children; h Institute of Health Policy, Management and Evaluation, University of Toronto, Toronto; i Department of Health Research Methods, Evidence, and Impact, McMaster University, Ontario, Canada; j Department of Orthopedic Surgery, SMG-SNU Boramae Medical Center; k Institute of Medical and Biological Engineering, Medical Research Center; l Department of Orthopedic Surgery, College of Medicine, Seoul National University, Seoul, Republic of Korea; m Division of Psychology and Mental Health, Faculty of Biology, Medicine and Health, University of Manchester, Manchester, UK; n Department of Biostatistics, Mailman School of Public Health, Columbia University, New York, New York, USA

**Keywords:** epidemiology, fracture, musculoskeletal disorder, orthopedics, public health

## Abstract

**Background:**

Population data that examines recent national trends in the prevalence of fractures are lacking in the United States (US).

**Materials and Methods:**

Analyses were based on 1999–2020 data from the National Health and Nutrition Examination Survey (NHANES). Primary outcomes included the prevalence of hip, wrist, and vertebral fractures among adults aged greater than or equal to 50 years. Changes in the prevalence over time were determined by joinpoint regression analysis. The authors also described the variation by fracture subtypes, sociodemographic characteristics, and their combination.

**Results:**

For adults aged greater than or equal to 50 years in NHANES 2017–March 2020, the authors estimated that there was 2.6 million Americans with hip fractures, 14.6 million Americans with wrist fractures, and 5.2 million Americans with vertebral fractures. The prevalence of wrist fractures significantly increased from 8.7% (7.4–9.9%) in 1999–2000 to 12.8% (11.6–14.1%) in 2017–March 2020 among adults aged greater than or equal to 50 years (*P* for trend=0.04); significant increases were also observed in fractures that occurred at age less than 50 years, non-Hispanic White, high family income groups, and several combination subgroups (e.g. fractures occurred at age <50 years among women). The prevalence of vertebral fractures increased from 2.2% (1.7–2.8%) in 1999–2000 to 4.6% (3.7–5.5%) in 2017–March 2020 among adults aged greater than or equal to 50 years (*P* for trend=0.02); significant increases were also observed in 50–64 years, women, non-Hispanic White, high family income groups and several combination subgroups (e.g. fractures that occurred at age <50 years among women). The authors did not observe significant trend changes in the prevalence of hip fractures among adults aged greater than or equal to 50 years between 1999 and 2020.

**Conclusion:**

The estimated prevalence of wrist and vertebral fractures significantly increased among US adults aged greater than or equal to 50 years from 1999 to 2020, although hip fractures did not significantly change.

## Introduction

Highlights

- The prevalence of wrist fractures increased by 50%, and the prevalence of vertebral fractures doubled, while the prevalence of hip fractures did not change in the United States between 1999 and 2020.
- The trends in the prevalence of wrist and vertebral fractures in subgroups were complex.
- The prevalence of hip fractures in the age of greater than or equal to 65 years was two times higher than that in the age of 50–64 years.
- The prevalence of wrist and vertebral fractures in non-Hispanic White was 2–4 times higher than that in Mexican American, non-Hispanic Black, and non-Hispanic Asian.

With a substantial impact on disability, morbidity, and mortality, fractures place an enormous burden on healthcare systems^1–6^. Although some previous studies reported estimates of hip, wrist, and vertebral fractures (e.g. 3.0% of the US older population had a hip fracture in 2005–2010^7^, 12.0% of the US adults ≥50 years had a wrist fracture in 2017–2018^8^, and 5.4% of the US adults ≥40 years had a vertebral fracture in 2013–2014^9^), no formal trend analyses were performed. Therefore, it was unclear whether the prevalence had changed over the years. In addition, most of the previous studies did not provide recent estimates of these fractures, ignored some important subgroups (e.g. non-Hispanic Asian and socioeconomic status), and had methodological limitations (e.g. estimates were not weighted or adjusted for design variables, potentially leading to biased results)^10^. Prevalence and prevalence trends of fractures at other sites (e.g. upper arm and ankle) are also important, but no relevant studies were identified.

Using recent data from the National Health and Nutrition Examination Survey (NHANES), this study aimed to provide up-to-date national estimates and evaluate trends in the prevalence of fractures among US adults. We also described the variation by fracture subtypes, sociodemographic characteristics, and their combination.

## Materials and methods

### Study population and sample

NHANES is a continuous and biennial publicly released dataset that provides information on the noninstitutionalized civilian population of the US. Data collected from in-home personal interviews have been released in 2-year cycles except for one 3.2-year cycle due to the influence of COVID-19^11^. Considering the available data on fractures, eight cycles (1999–2000, 2001–2002, 2003–2004, 2005–2006, 2007–2008, 2009–2010, 2013–2014, 2017–March 2020) were included in this study. Adults aged greater than or equal to 20 years were included.

As a secondary analysis of anonymized data, this study did not involve human participants. Thus, informed consent and institutional review board approval were not required. Our study has been reported in line with the strengthening the reporting of cohort, cross-sectional and case–control studies in surgery (STROCSS) criteria^12^.

## Statistical analysis

### Primary outcomes

Participants reported fractures after receiving doctors’ fracture diagnoses. Primary outcomes included the prevalence of hip, wrist, and vertebral fractures among adults aged greater than or equal to 50 years with a comprehensive collection of fracture data.

The prevalence of hip, wrist, and vertebral fractures was estimated from the most recent NHANES cycle (2017–March 2020). Following the endorsement from Lesko *et al.*^13^, the prevalence estimates were reported as unadjusted. Analyses were further stratified by subgroups including subtypes of fractures, socioeconomic characteristics, and their combination.

Subtypes of fractures included fragility fractures (≥50 years), nonfragility fractures (≥50 years), and fractures (<50 years) (details in Appendix S1 in Supplementary File 1, Supplemental Digital Content 1, http://links.lww.com/JS9/B265).

We included the following socioeconomic characteristics: age (middle-aged 50–64 and older adults ≥65 years)^14,15^, sex (men and women), race and ethnicity (all Hispanic, Mexican American, non-Hispanic White, non-Hispanic Black, non-Hispanic Asian, and other races), and family income level [the ratio of family income to poverty (PIR) as ≤1.30, 1.30< PIR ≤3.50, and PIR >3.50]^16^.

To assess the relative risk within each socioeconomic subgroup in the stratified analysis, logistic regression was performed with a two-step modeling framework: step 1, unadjusted analyses; and step 2, multivariable analyses adjusted for all socioeconomic variables. Odds ratios and 95% CIs were obtained. Relative risk within subtypes of fractures was not assessed because the overlap existed between each subtype of fractures.

We used a relative percentage change per 2-year cycle with a 95% CI to determine changes in the prevalence of fractures over time. Based on the analytic guidelines of joinpoint software, we used joinpoint regressions with heteroscedastic and uncorrelated errors for outcomes including at least seven cycles^17–19^. We chose the minimum number of joinpoints (0 joinpoint, which is a straight line) to avoid potential overfitting. Following the framework the previous study employed, trends in the prevalence of fractures were analyzed in each NHANES cycle^20^. Trends in the prevalence of fractures were further stratified on variables of interest. Based on the analytic guidelines, the trends in the prevalence of fractures among all Hispanic between 2007 and 2020 (four cycles) were analyzed because these were oversampled since 2007^21^. Similarly, non-Hispanic Asians were oversampled from 2011, including only two cycles (NHANES 2013–2014 and 2017–2020)^21^. For all Hispanic and non-Hispanic Asians that included less than seven data points, absolute differences and relative percentage changes with 95% CIs were estimated using *t* tests^22^.

### Secondary outcomes

Secondary outcomes included the prevalence of fractures at other locations among adults aged greater than or equal to 50 years and each type of fractures among adults aged 20–49 years. We were not able to analyze the secondary outcomes in the same detail as the primary outcomes (detailed reasons are in Appendix S2 in Supplementary File 1, Supplemental Digital Content 1, http://links.lww.com/JS9/B265). To provide as comprehensive information as possible, we used available data to conduct the following simplified analyses of secondary outcomes: absolute differences and percentage changes in the prevalence of hip, wrist, and vertebral fractures between 2009–2010 and 1999–2000 and fractures at other locations between 2009–2010 and 2005–2006 for adults aged 20–49 years, and the prevalence of fractures at other locations between 2017–March 2020 and 2005–2006 for adults aged greater than or equal to 50 years with 95% CIs were analyzed using Welch’s *t*-test.

### Additional analyses

We also performed the following three additional analyses. First, based on the reviewer’s suggestion, we performed a comprehensive search for fractures and osteoporosis guidelines from the past 10 years through the MEDLINE database via Ovid. The search strategy was described in Appendix S3 in Supplementary File 1 (Supplemental Digital Content 1, http://links.lww.com/JS9/B265). Second, the results of the comprehensive search showed that recent guidelines recommend the use of antiosteoporotic drugs to prevent osteoporotic fractures in adults aged greater than or equal to 50 years or postmenopausal women. In our study, we found that the prevalence of fragility fractures (≥50 years) did not change among adults aged greater than or equal to 50 years. To explore the potential role of the use of antiosteoporotic drugs in our study, we analyzed the prevalence trends of the use of antiosteoporotic drugs (bisphosphonates, nonbisphosphonates, and both) based on the framework of Carlos and Orces^23^. Last, the results of the comprehensive search showed that there were insufficient guidelines on the prevention of fractures for adults aged less than 50 years at low risk of fractures. We found that the prevalence of wrist and vertebral fractures (<50 years) was significantly increased among women but not in men. To explore the possible reasons for the sex difference, we further analyzed the prevalence trends by age of first fracture occurrence [0–2 years (infants and toddlers), 3–5 years (preschoolers), 6–11 years (middle childhood), 12–19 years (teenagers), 0–19 years (overall children), 20–29 years, 30–39 years, 40–49 years, and 20–49 years) and sex according to the age range of stage of life^24^.

### Other analysis details

Based on the recommendations from NHANES Survey Methods and Analytic Guidelines, weights and design variables were included to obtain unbiased results^25^. A complete case analysis was performed for the main analyses. Data were analyzed and visualized with R version 4.2.0 with haven, tidyverse, Hmisc, survey, naniar, and ggplot2 packages and Joinpoint Regression Program version 4.9.0.1^17,18^.

## Results

In total, we included 23 604 participants aged greater than or equal to 50 years from 1999–March 2020, with a weighted mean age of 63.7 (standard error=0.1) years, 46.3% were men, 4.5% Mexican American, 75.7% non-Hispanic White, and 9.7% non-Hispanic Black. We also included 16 654 participants aged 20–49 years from 1999–2010, with a weighted mean age of 34.8 (standard error=0.1) years, 49.2% were men, 10.1% Mexican American, 65.3% non-Hispanic White, and 12.4% non-Hispanic Black.

## Prevalence of hip fractures among adults aged greater than or equal to 50 years

During the period 2017–March 2020, we estimated that there were 2.6 million Americans with hip fractures with a prevalence of 2.3% (1.5–3.0%) (Table 1). Multivariable adjusted prevalence of hip fractures was significantly higher in participants aged greater than or equal to 65 years than 50–64 years (3.6 vs. 1.2%, *P*=0.02) (Tables 1 and 2). No significant differences were found between other subgroups (Tables 1 and 2).

**Table 1:** Prevalence of hip, wrist, and vertebral fractures among US adults aged greater than or equal to 50 years, 2017–March 2020.

		Hip fractures		Wrist fractures		Vertebral fractures	
Characteristics	Total No.	No.	Prevalence, % (95% CI)	No.	Prevalence, % (95% CI)	No.	Prevalence, % (95% CI)
Overall prevalence	4987	117	2.3 (1.5–3.0)	519	12.8 (11.6–14.1)	180	4.6 (3.7–5.5)
Socioeconomic subgroups							
Age group, years							
50–64	2576	33	1.2 (0.4–2.1)	248	11.9 (10.0–13.9)	89	4.0 (2.9–5.1)
≥65	2411	84	3.6 (2.6–4.6)	271	13.9 (12.3–15.5)	91	5.4 (4.1–6.6)
Sex							
Men	2465	47	2.1 (0.9–3.2)	260	13.0 (11.1–14.9)	97	4.5 (3.2–5.7)
Women	2522	70	2.5 (1.7–3.3)	259	12.7 (10.8–14.6)	83	4.7 (3.4–6.0)
Race and ethnicity							
All Hispanic	964	11	1.1 (0.4–1.9)	83	8.3 (5.3–11.3)	27	3.2 (1.9–4.5)
Mexican American	457	3	0.7 (0.0–1.6)^a^	30	6.9 (2.4–11.4)	11	2.1 (1.0–3.3)
Non-Hispanic White	1964	73	2.7 (1.7–3.7)	289	15.0 (13.3–16.7)	104	5.2 (4.0–6.5)
Non-Hispanic Black	1333	24	1.7 (0.8–2.6)	97	6.6 (5.4–7.9)	23	1.7 (1.0–2.4)
Other races including non-Hispanic Asian^b^	726	9	1.3 (0.5–2.1)	50	8.3 (4.9–11.6)	26	4.6 (1.7–7.5)
Non-Hispanic Asian	530	6	1.3 (0.4–2.3)	23	4.3 (2.3–6.2)	12	2.6 (1.3–3.8)
Family income level							
PIR≤1.30	1085	30	2.4 (1.5–3.2)	108	11.1 (8.7–13.5)	34	3.3 (2.0–4.6)
1.30< PIR≤3.50	1711	43	2.9 (1.7–4.2)	171	13.4 (10.9–15.9)	68	5.3 (3.6–6.9)
PIR>3.50	1413	29	1.8 (0.6–3.0)	158	13.5 (11.4–15.5)	51	4.9 (3.2–6.6)
Subtypes of fractures subgroups							
Fragility fractures (≥50 years)	161	42	0.8 (0.5–1.0)	115	2.2 (1.7–2.6)	19	0.4 (0.1–0.7)
Nonfragility fractures (≥50 years)	155	37	0.6 (0.3–0.9)	86	1.8 (1.2–2.3)	41	0.9 (0.5–1.3)
Fractures (<50 years)	459	36	0.7 (0.4–1.1)	349	9.8 (8.6–11.0)	111	3.0 (2.2–3.9)

PIR, the ratio of family income to poverty.

a Lower end of 95% CI was smaller than 0.0 which was not accurate because of the small sample size, thus the value was recorded as 0.0.

b Other races including non-Hispanic Asians, multiracial, and other than Hispanic, non-Hispanic White, and non-Hispanic Black.

**Table 2:** Logistic regression analysis of hip, wrist, and vertebral fractures among US adults aged greater than or equal to 50 years, 2017–March 2020.

		Hip fractures		Wrist fractures		Vertebral fractures	
Variables	Adjusted or not	OR (95% CI)	*P*	OR (95% CI)	*P*	OR (95% CI)	*P*
Age, years							
50–64		1.00 (reference)		1.00 (reference)		1.00 (reference)	
≥65	Unadjusted	2.95 (1.58–5.50)	0.002	1.19 (0.94–1.50)	0.15	1.36 (0.98–1.91)	0.08
	Multivariable adjusted	2.37 (1.21–4.64)	0.02	1.05 (0.78–1.41)	0.75	1.28 (0.84–1.94)	0.26
Sex							
Men		1.00 (reference)		1.00 (reference)		1.00 (reference)	
Women	Unadjusted	1.19 (0.67–2.14)	0.56	0.97 (0.75–1.26)	0.83	1.05 (0.70–1.58)	0.82
	Multivariable adjusted	0.94 (0.56–1.59)	0.82	0.98 (0.73–1.31)	0.87	1.05 (0.68–1.62)	0.83
Race and ethnicity							
Non-Hispanic White		1.00 (reference)		1.00 (reference)		1.00 (reference)	
Mexican American	Unadjusted	0.27 (0.07–1.07)	0.08	0.42 (0.20–0.88)	0.03	0.40 (0.21–0.75)	0.01
	Multivariable adjusted	0.36 (0.09–1.46)	0.17	0.46 (0.20–1.04)	0.08	0.43 (0.22–0.86)	0.03
Non-Hispanic Black	Unadjusted	0.62 (0.31–1.21)	0.18	0.40 (0.32–0.51)	< 0.001	0.31 (0.20–0.51)	< 0.001
	Multivariable adjusted	0.67 (0.34–1.30)	0.25	0.38 (0.30–0.49)	< 0.001	0.25 (0.13–0.48)	< 0.001
Non-Hispanic Asian	Unadjusted	0.50 (0.23–1.07)	0.09	0.25 (0.16–0.41)	< 0.001	0.48 (0.29–0.80)	0.01
	Multivariable adjusted	0.67 (0.30–1.51)	0.35	0.27 (0.15–0.49)	< 0.001	0.54 (0.30–0.96)	0.051
Other excluding non-Hispanic Asian	Unadjusted	0.48 (0.20–1.17)	0.12	0.90 (0.54–1.50)	0.69	1.44 (0.53–3.90)	0.48
	Multivariable adjusted	0.39 (0.11–1.35)	0.16	0.97 (0.58–1.60)	0.90	1.76 (0.63–4.94)	0.30
Family income level							
PIR≤1.30		1.00 (reference)		1.00 (reference)		1.00 (reference)	
1.30< PIR≤3.50	Unadjusted	1.25 (0.69–2.27)	0.47	1.24 (0.88–1.74)	0.23	1.63 (1.00–2.66)	0.06
	Multivariable adjusted	0.94 (0.51–1.74)	0.85	1.06 (0.74–1.51)	0.77	1.42 (0.89–2.25)	0.16
PIR>3.50	Unadjusted	0.77 (0.33–1.79)	0.55	1.24 (0.97–1.60)	0.11	1.52 (0.84–2.75)	0.18
	Multivariable adjusted	0.62 (0.29–1.30)	0.22	1.01 (0.78–1.30)	0.95	1.33 (0.77–2.30)	0.31

OR, odds ratio; PIR, the ratio of family income to poverty.

**Figure 1:**
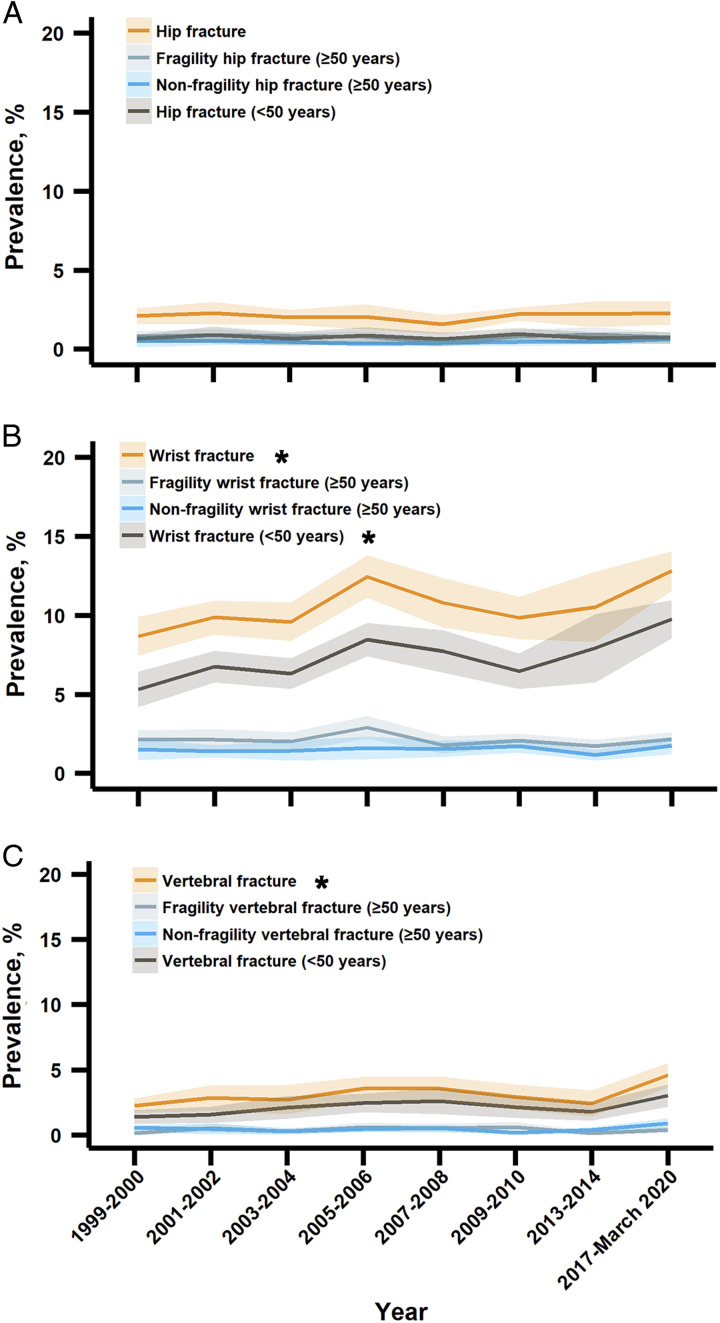
Trends in the prevalence of hip, wrist, and vertebral fractures among US adults aged greater than or equal to 50 years by overall and subtypes of fractures, 1999–March 2020. * The prevalence trend was significantly increased.

**Figure 2:**
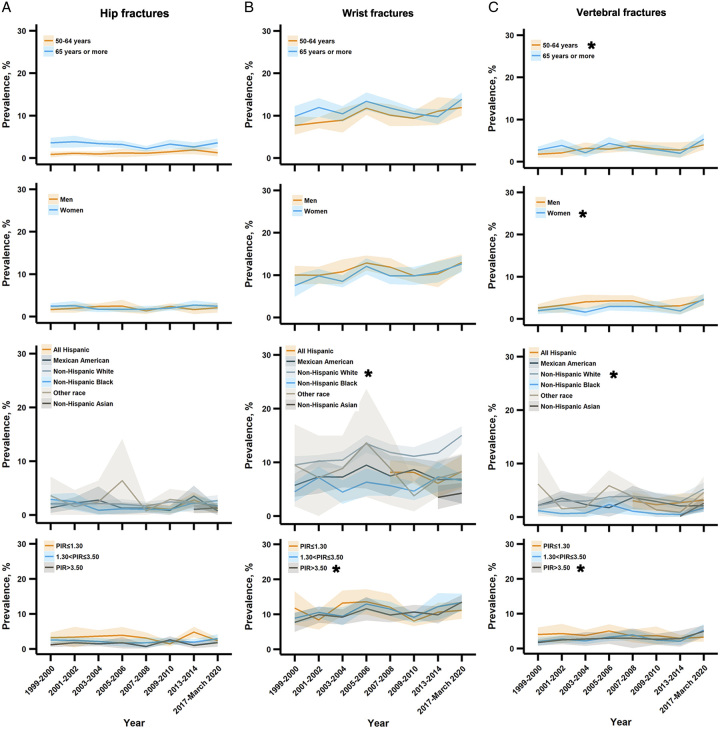
Trends in the prevalence of hip, wrist, and vertebral fractures among US adults aged greater than or equal to 50 years by socioeconomic characteristics, 1999–March 2020. PIR = the ratio of family income to poverty. *The prevalence trend was significantly increased.

**Figure 3:**
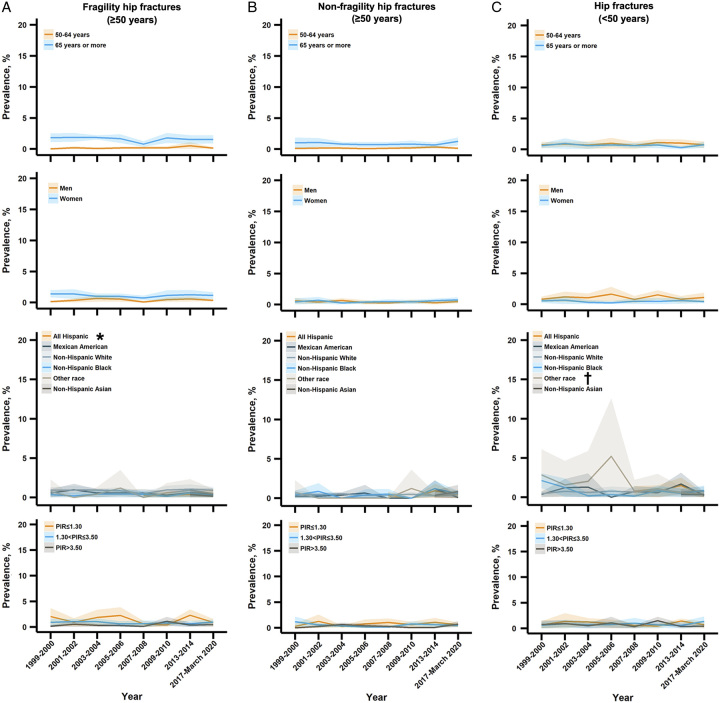
Trends in the prevalence of hip fractures among US adults aged greater than or equal to 50 years by combination subgroups, 1999–March 2020. PIR = the ratio of family income to poverty. *The prevalence trend was significantly increased. ^†^The prevalence trend was significantly decreased.

We did not observe significant trend changes in the overall prevalence of hip fractures from 2.1% (1.6–2.6%) in 1999–2000 to 2.3% (1.5–3.0%) in 2017–2020 (*P* for trend=0.56) (Fig. 1). However, in subgroup analyses (Figs. 1–3), we found that the prevalence of hip fractures (<50 years) significantly decreased among other races including non-Hispanic Asians (*P* for trend=0.007) (Fig. 3).

## Prevalence of wrist fractures among adults aged greater than or equal to 50 years

From 2017–March 2020, we estimated that there were 14.6 million Americans with wrist fractures with a prevalence of 12.8% (11.6–14.1%) (Table 1). Multivariable adjusted prevalence of wrist fractures was significantly higher in non-Hispanic White than non-Hispanic Black (15.0 vs. 6.6%, *P*<0.001) and non-Hispanic Asian (15.0 vs. 4.3%, *P* <0.001) groups (Tables 1 and 2). Significant differences were not observed among other subgroups (Tables 1 and 2).

**Figure 4:**
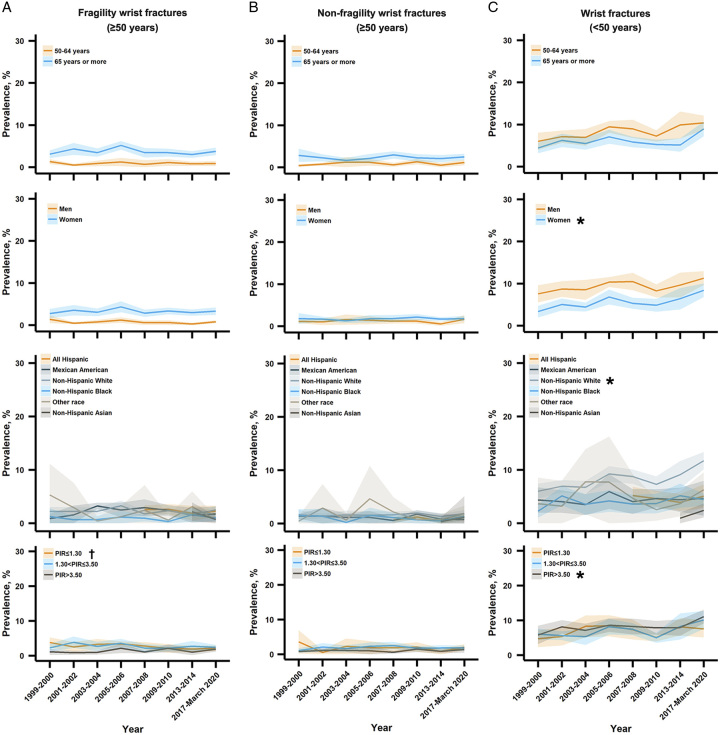
Trends in the prevalence of wrist fractures among US adults aged greater than or equal to 50 years by combination subgroups, 1999-March 2020. PIR = the ratio of family income to poverty. *The prevalence trend was significantly increased. ^†^The prevalence trend was significantly decreased.

Between 1999 and 2020, the estimated overall prevalence of wrist fractures significantly increased from 8.7% (7.4–9.9%) to 12.8% (11.6–14.1%), with a relative increase of 3.1% (0.2–6.1%), *P* for trend=0.04 (Fig. 1). In subgroup analyses, significant increases in the prevalence trends were observed in wrist fractures (<50 years), non-Hispanic White, higher family income (PIR >3.50), and combination subgroups including [i.e. wrist fractures (<50 years) increased among women, non-Hispanic White, and higher family income (PIR >3.50) subgroups] (all *P* for trend <0.05) (Figs. 1, 2, and 4). A significantly decreasing trend in the prevalence of fragility wrist fractures (≥50 years) was observed among the lowest family income (PIR ≤1.30) subgroup (*P* for trend=0.01) (Fig. 4).

## Prevalence of Vertebral Fractures among adults aged greater than or equal to 50 years

From 2017–March 2020, we estimated that there were 5.2 million Americans with vertebral fractures with a prevalence of 4.6% (3.7–5.5%) (Table 1). Multivariable adjusted prevalence of vertebral fractures was significantly higher in non-Hispanic White than Mexican American (5.2 vs. 2.1%, *P*=0.03) and non-Hispanic Black (5.2vs. 1.7%, *P* <0.001) groups (Tables 1 and 2). There were no significant differences between other subgroups (Tables 1 and 2).

**Figure 5:**
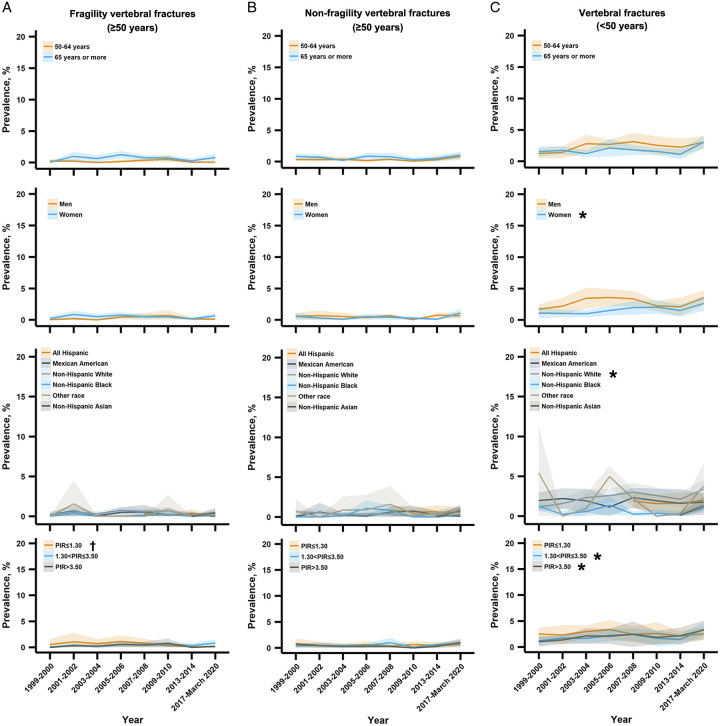
Trends in the prevalence of vertebral fractures among US adults aged greater than or equal to 50 years by combination subgroups, 1999–March 2020. PIR = the ratio of family income to poverty. *The prevalence trend was significantly increased. ^†^The prevalence trend was significantly decreased.

The estimated overall prevalence of vertebral fractures significantly increased from 2.2% (1.7–2.8%) in 1999–2000 to 4.6% (3.7–5.5%) in 2017–March 2020, with a relative increase of 6.0% (1.4–10.8%) (*P* for trend=0.02) per cycle (Fig. 1). In subgroup analyses, significant increases in the prevalence trends were found in 50–64 years, women, non-Hispanic White, higher family income subgroups, and several combination subgroups [i.e. vertebral fractures (<50 years) significantly increased among women, non-Hispanic White, and higher family income (1.30< PIR ≤3.50, PIR >3.50) groups], all *P* for trend <0.05 (Figs. 2 and 5). The prevalence of fragility vertebral fractures (≥50 years) significantly decreased among the lowest family income (PIR ≤1.30) subgroup (*P* for trend=0.008) (Fig. 5). In addition, we found that the prevalence of vertebral fractures among non-Hispanic Asians was increased in 2017–2020 [2.6% (1.3–3.8%)] compared with 2013–2014 [0.2% (0.0–0.4%)] with a mean difference of 2.4% (1.1–3.7%) and a relative change of 1555.5% (728.6–2382.5%) (Fig. 2 and Appendix S4 in Supplementary File 1, Supplemental Digital Content 1, http://links.lww.com/JS9/B265).

## Prevalence of fractures at other locations among adults aged greater than or equal to 50 years

Between 2005 and 2020, the overall estimated prevalence of fractures at other locations among adults aged greater than or equal to 50 years did not significantly change from 31.3% (28.8–33.9%) in 2005–2006 to 28.1% (26.0–30.1%) in 2017–March 2020 with percentage changes of −10.5% (−21.0–0.03%) (Appendix S5 in Supplementary File 1, Supplemental Digital Content 1, http://links.lww.com/JS9/B265). In subgroup analyses, significant decrease in the prevalence was observed in nonfragility fractures at other locations, fragility knee fractures, 50–64 years, and other race groups (Appendix S5 in Supplementary File 1, Supplemental Digital Content 1, http://links.lww.com/JS9/B265).

## Prevalence of fractures among adults aged 20–49 years

Between 1999 and 2010, the overall prevalence of hip fractures did not significantly change from 0.4% (0.1–0.8%) in 1999–2000 to 0.6% (0.4–0.9%) in 2009–2010 with percentage changes of 51.3% (−48.6–151.2%) among adults aged 20–49 years (Appendix S6 in Supplementary File 1, Supplemental Digital Content 1, http://links.lww.com/JS9/B265). However, in subgroup analyses, we found that the prevalence of hip fractures significantly increased in 35–49 years and women groups. The estimated overall prevalence of wrist fractures significantly decreased from 10.1% (8.7–11.5%) in 1999–2000 to 7.9% (6.5–9.3%) in 2009–2010, with percentage changes of −21.9% (−41.3–−2.5%) (Appendix S6 in Supplementary File 1, Supplemental Digital Content 1, http://links.lww.com/JS9/B265). In subgroup analyses, significant decreases in the prevalence were observed in 35–49 years, men, Mexican American, and middle family income (1.30< PIR ≤3.50) groups. However, no significant change was found in the estimated prevalence of vertebral fractures overall and by subgroup (Appendix S6 in Supplementary File 1, Supplemental Digital Content 1, http://links.lww.com/JS9/B265). Between 2005 and 2010, the overall estimated prevalence of fractures at other locations among adults aged 20–49 years did not significantly change from 18.0% (15.7–20.4%) in 2005–2006 to 16.8% (15.1–18.5%) in 2009–2010 with percentage changes of −6.9% (−23.1–9.3%) (Appendix S7 in Supplementary File 1, Supplemental Digital Content 1, http://links.lww.com/JS9/B265). However, in subgroup analyses, we found a significant decrease in the prevalence of fragility fractures at other locations, including toe fractures (Appendix S7 in Supplementary File 1, Supplemental Digital Content 1, http://links.lww.com/JS9/B265).

## Results from additional analyses

A total of 9014 references were identified through the search. Finally, 31 guidelines from 12 countries were included in which the grade of the recommendation or the quality of the evidence was included [Appendix S3 in Supplementary File 1, Supplemental Digital Content 1, http://links.lww.com/JS9/B265 (simple version) and Supplementary File 2, Supplemental Digital Content 2, http://links.lww.com/JS9/B266 (full version)]. The 12 countries included Argentina, Brazil, France, India, Italy, Poland, Russia, Saudi Arabia, Scotland, Spain, UK, and the US. Among them, there were eight records about the management of fractures, 23 records about the management of osteoporosis among adults aged greater than or equal to 50 years and postmenopausal women at moderate or high risk of fractures, and only two records mentioned the prevention of fractures for adults aged less than 50 years at low risk of fractures (details in Appendix S3 in Supplementary File 1, Supplemental Digital Content 1, Supplemental Digital Content 1, http://links.lww.com/JS9/B265)^26,27^.

Regarding the potential role of the use of antiosteoporotic drugs, we found that the prevalence of antiosteoporotic drugs use peaked [11.2% (9.1–13.3%)], and the prevalence of fragility hip fractures reached a minimum [0.8% (0.3–1.4%)] in 2007–2008. After that, the prevalence of antiosteoporotic drug use seemed to decrease, and the prevalence of fragility hip fractures seemed to increase (Appendix S8 in Supplementary File 1, Supplemental Digital Content 1, http://links.lww.com/JS9/B265).

Considering the sex differences in the prevalence trends of wrist and vertebral fractures before the age of 50 years, we found significant increases in the prevalence of first wrist fractures occurring before the age of 30 years (preschoolers, teenagers, and adults aged 20–29 years) and first vertebral fractures occurring before the age of 20 years (Appendix S9 in Supplementary File 1, Supplemental Digital Content 1, http://links.lww.com/JS9/B265) among females. However, the prevalence of wrist and vertebral fractures that occurred at each age range did not significantly change among males (Appendix S9 in Supplementary File 1, Supplemental Digital Content 1, http://links.lww.com/JS9/B265).

## Discussion

### Principal findings

We provided up-to-date trends in the prevalence of hip, wrist, and vertebral fractures among US adults aged greater than or equal to 50 years. Between 1999 and 2020, the estimated prevalence of wrist fractures increased from 8.7 to 12.8% by ~50%, and the estimated prevalence of vertebral fractures doubled from 2.2 to 4.6%, while the estimated prevalence of hip fractures did not significantly change over time.

The trends varied across subgroups. Interestingly, wrist and vertebral fractures before the age of 50 years increased in women, non-Hispanic White, and higher family income subgroups. On the brighter side, the fragility fractures of the wrist and vertebras after the age of 50 years significantly decreased in the lower family income subgroup.

## Comparison with previous studies

Our study included methodological improvements, a series of NHANES data and specific causes of fractures to provide a reliable prevalence and prevalence trends of hip, wrist, and vertebral fractures in US adults aged greater than or equal to 50 years. Three previous studies used specific NHANES fracture data to estimate: 1) the prevalence of hip fractures among adults greater than or equal to 65 years with 2005–2010 data without adjusting weights and design variables^7^; 2) the prevalence of wrist fractures among adults greater than or equal to 50 years with 2013–2014 and 2017–2018 data^8^; 3) the prevalence of vertebral fractures among adults greater than or equal to 40 years using 2013–2014 data^9^. We supplemented the previous studies with all available data points, fractures at other locations, and fractures among adults aged less than 50 years. Additionally, our study was the most ethnically inclusive and the first time examined the prevalence of fractures among non-Hispanic Asians.

The prevalence of fragility fractures among adults aged greater than or equal to 50 years did not significantly change in this study. Antiosteoporotic drugs are effective treatments recommended by guidelines^28^. Based on available data, we found the prevalence of antiosteoporotic drug use peaked, and the prevalence of fragility hip fractures reached a minimum in 2007–2008, which may imply that antiosteoporotic drug plays an important role in reducing the risk of fragility hip fractures. After that, the prevalence of antiosteoporotic drug use seemed to decrease, and the prevalence of fragility hip fractures seemed to increase. Although available data may be insufficient to detect statistical significance, this phenomenon may warrant attention and future data collection on fracture prevalence should be continued.

The prevalence trends of wrist and vertebral fractures (<50 years) significantly increased among women but not in men aged greater than or equal to 50 years. The increased trends were observed for first wrist fractures occurring before the age of 30 years and first vertebral fractures occurring before the age of 20 years, that is, these fractures occurred approximately between 1923 and 2000 (80 years old in 2000 is equivalent to 3 years old in 1923, and 50 years old in 2020 is equivalent to 30 years old in 2000). A population-based study investigated the incidence of distal forearm fractures among US residents aged less than 35 years between 1969 and 2001 (1969–1971, 1979–1981, 1989–1991, and 1999–2001)^29^. The results showed that the incidence rates (per 100 000 person-years) for distal forearm fractures generally increased in females aged less than 30 years (566.1 to 832.3 in 5–9 years, 490.9 to 799.5 in 10–14 years, 119.5 to 221.3 in 15–19 years, 10.6 to 114.6 in 20–24 years)^29^. Of these fractures in both sexes, sports injury was the only cause of fractures with an increased proportion (from 48.0 to 64.3% among males; from 37.2 to 55.8% among females)^29^. Based on the 2021–2022 High School Athletics Participation Survey conducted by the National Federation of State High School Associations, the number of girls participants increased ninefold between 1971–1972 and 1999–2000 (from 294 015 to 2 675 874), while the number of boys participants did not obviously increase (from 3 666 917 to 3 861 749)^30^. Therefore, we speculate that the increased prevalence of wrist fractures in women under 30 in this study is mainly due to the increase in sports injuries caused by the rapid increase in the number of female sports participants. More attention needs to be paid to establishing guidelines for the neglected population at low risk of osteoporotic fractures.

Although the prevalence of wrist fractures occurring before the age of 20 did not change among adults aged 20–49 years (e.g. from 5.8 to 6.0% between 1999 and 2010), the value was higher than that among adults aged greater than or equal to 50 (e.g. from 1.3 to 4.8% between 1999 and 2020) (Appendix S9 in Supplementary File 1, Supplemental Digital Content 1, http://links.lww.com/JS9/B265), which means that wrist fractures in nonadults had risen to a stable level by 2010. It is worth noting that the sports participation rate of children has been slightly rising between 2012 and 2021^31^. However, data on wrist fractures among adults aged 20–49 years was not collected during this period, preventing us from understanding subsequent trends in the prevalence.

We found no supporting information on the reasons for the increased prevalence of vertebral fractures occurring before the age of 20 years. Based on a large national sample of US high school athletes between 2005–2006 and 2012–2013, sports-related compression fractures were most common in girls’ cross country (10.6 per 100 000) and girls’ gymnastics (7.4 per 100 000), with the lower back/lumbar spine/pelvis (15.2%) being the third most common site^32^. Therefore, considering the increase in the number of girls participants, we speculated that sports-related lumbar vertebral stress fractures might be one of the reasons for the increase in vertebral fractures occurring before the age of 20 among women in this study.

The prevalence of wrist and vertebral fractures and both fractures (<50 years) increased among non-Hispanic White but not among the remaining ethnic groups. It could be due to a slighter decline in smoking among non-Hispanic White compared to other race and ethnicity groups^33,34^, or due to a significant decline in bone mineral density among non-Hispanic White men with no change among other races and ethnicities^35^.

Considering the increased prevalence of wrist and vertebral fractures and both fractures (<50 years) among the highest family income subgroup, we did not find plausible supporting evidence, which needs to be elucidated by relevant studies in the future.

For the first time, we examined the prevalence of fractures among the US non-Hispanic Asian group aged greater than or equal to 50 years. We found that the prevalence of vertebral fractures is likely increasing (e.g. from 2013 to 2020, vertebral fractures increased from 0.2 to 2.6%) maybe due to an insufficient number of events, which requires further investigations using combined cycles to confirm in the future. The prevalence of nonfragility fractures at other locations was also found to be increased among adults aged greater than or equal to 50 years. The specific reasons for the increased prevalence need to be further investigated in the future.

## Limitations

Our study has several limitations. First, although NHANES data were collected from 1999 to 2020, we did not include two cycles (2011–2012 and 2015–2016) due to the lack of data on fracture data. Second, the 2013–2014 and 2017–March 2020 cycles were not included in the adults aged 20–49 years analyses, as information on fractures in 2013–2014 was recorded for participants older than 40 years and in 2017–2020 for participants older than 50 years. Information on fractures at other locations were not collected before 2005. Third, although a fracture is a major health event unlikely to be subject to recall bias, all types of fractures in this study were self-reported. As the self-reported question is imperfect concerning specificity and sensitivity, a misclassification bias cannot be completely ruled out^36–38^. Fourth, specific causes of fractures that occurred before the age of 50 years were not collected, which prevented us from identifying the detailed reasons for the increasing prevalence trends of wrist and vertebral fractures (<50 years) and whether this has changed after 2010. In the future, it is necessary to focus on collecting the causes of fractures occurring at all ages, which will provide meaningful data for the development of fracture prevention guidelines for low risk populations. Fifth, the prevalence trends of fractures among non-Hispanic Asians could not be analyzed because data were collected in only two cycles. However, we found that vertebral fractures might have an increasing trend through available data, which needs to be confirmed by continuing to collect data in the future. Last, due to the differences in the ethnic composition between the US and other countries, the findings of our study cannot be generalized to populations from countries other than US^39–41^. Therefore, readers from other countries need to interpret out results with caution.

## Implications for practice and researchers

Our study fills an essential gap in understanding the latest prevalence of fractures and their prevalence trends in the past two decades among US adults with implications for practice, policy, and further research. We indicated that fracture prevention could be personalized according to population subgroups (e.g. ethnic groups). For fracture prevention, current guidelines focus on overall adults, women aged greater than or equal to 65 years, postmenopausal women, or men aged greater than or equal to 50 years^27,42–45^. Based on our results, future guidelines should include content about identified personalized prevention, especially low risk population. Given the fracture burden, these findings highlight that wrist and vertebral fractures among adults aged greater than or equal to 50 years, especially wrist fractures that occurred before the age of 50, are significant healthcare problems requiring more attention and better policies.

## Conclusion

Based on NHANES data, the estimated prevalence of wrist and vertebral fractures significantly increased among US adults aged greater than or equal to 50 years from 1999 to 2020, while in the same period the estimated prevalence of hip fractures did not significantly change. Therefore, wrist and vertebral fractures remain a significant public health concern that requires more attention for their burden to be reduced.

## Ethical approval

As a secondary analysis of anonymized data, this study did not involve human participants. Thus, informed consent and institutional review board approval were not required.

## Consent

As a secondary analysis of anonymized data, this study did not involve human participants. Thus, informed consent and institutional review board approval were not required.

## Sources of funding

Bin Xu was supported by the China Scholarship Council (no. 201809120013). The funding source had no role in designing, conducting, or reporting the study.

## Author contribution

B.X.: data curation, formal analysis, visualization, writing – original draft, writing – review and editing; M.R.R.: formal analysis, methodology, writing – original draft, writing – review and editing; D.B.A., B.S., L.Y., and Y.C.: writing – review and editing; S.F.: conceptualization, supervision, writing – review and editing; J.H.L.: conceptualization, supervision, writing – review and editing; L.C.: conceptualization, methodology, supervision, writing – review and editing.

## Conflicts of interest disclosure

There are no conflicts of interest.

## Research registration unique identifying number (UIN)

1. Name of the registry: not applicable.
2. Unique identifying number or registration ID: not applicable.
3. Hyperlink to your specific registration (must be publicly accessible and will be checked): not applicable.

## Guarantor

Lingxiao Chen.

## Data availability statement

Data are open access and can be downloaded at: https://wwwn.cdc.gov/Nchs/Nhanes/.

## Provenance and peer review

This paper was no invited.

## Supplementary Material
