# Supplementary material for: Trends in prevalence of fractures among adults in the United States, 1999–2020: a population-based study
Source: Int J Surg. 2023 Nov 3;110(2):721–32. doi: 10.1097/JS9.0000000000000883 (PMC10871608; doi:10.1097/JS9.0000000000000883)
Supplement: Supplementary file 1 [file js9-110-0721-s001.docx]

Trends in Prevalence of Fractures Among Adults in the United States, 1999-2020: A Population-Based Study

**Contents**

[Appendix S1. Definitions of subtypes of fractures 2](#_Toc145881557)

[Appendix S2. Reasons why secondary outcomes cannot be analysed in the same detail as the primary outcomes 3](#_Toc145881558)

[Appendix S3. Guidelines for fractures and osteoporosis published over the past 10 years 5](#_Toc145881559)

[Appendix S4. Prevalence of hip, wrist, and vertebral fractures and fractures at other locations among non-Hispanic Asian aged ≥50 years, 2013-2020 19](#_Toc145881560)

[Appendix S5. Prevalence of fractures at other locations among US adults aged ≥50 years, 2005-March 2020 21](#_Toc145881561)

[Appendix S6. Trends in the prevalence of hip, wrist, and vertebral fractures stratified by demographic characteristics among US adults aged 20-49 years, 1999-2010 27](#_Toc145881562)

[Appendix S7. Prevalence of fractures at other locations among US adults aged 20-49 years, 2005-2010 31](#_Toc145881563)

[Appendix S8. Trends in the prevalence of anti-osteoporotic drug use and fragility hip, wrist, and vertebral fractures among US adults aged ≥50 years, 1999-March 2020 35](#_Toc145881564)

[Appendix S9. Trends in the prevalence of wrist and vertebral fractures (<50 years) stratified by detailed age and sex among US adults aged ≥50 years, 1999-March 2020 37](#_Toc145881565)

## Appendix S1. Definitions of subtypes of fractures

Fragility fractures (≥50 years) resulted from falls when standing upright or lower than that (e.g., when tripped, slipped, or fell out of bed), while non-fragility fractures (≥50 years) included fractures due to hard falls (e.g., falling off a ladder, step stool, or down the stairs), car accidents, or other severe trauma that occurred after the age of 50 years.^1^ Fractures (<50 years) were defined as any fractures that occurred before the age of 50, as there were no sufficient details about the causes of fractures recorded before the age of 50 years. The same participants were asked about these three subtypes of fractures.

## Appendix S2. Reasons why secondary outcomes cannot be analysed in the same detail as the primary outcomes

We were not able to analyse the secondary outcomes in the same detail as the primary outcomes for the following three reasons: (1) fracture data for adults aged 20-49 years were only collected between 1999 and 2010 (6 cycles), which prevent us from providing the up-to-date prevalence and prevalence trends; (2) the subgroups collection methods for fractures at other locations were different from hip, wrist, and vertebral fractures (following figure); and (3) since the fractures at other locations were collected from 2005, the data were less than 7 cycles (3 cycles [2005-2010] among adults aged 20-49 years; 5 cycles [2005-March 2020] among adults aged ≥50 years), which were not suitable for trend analyses.


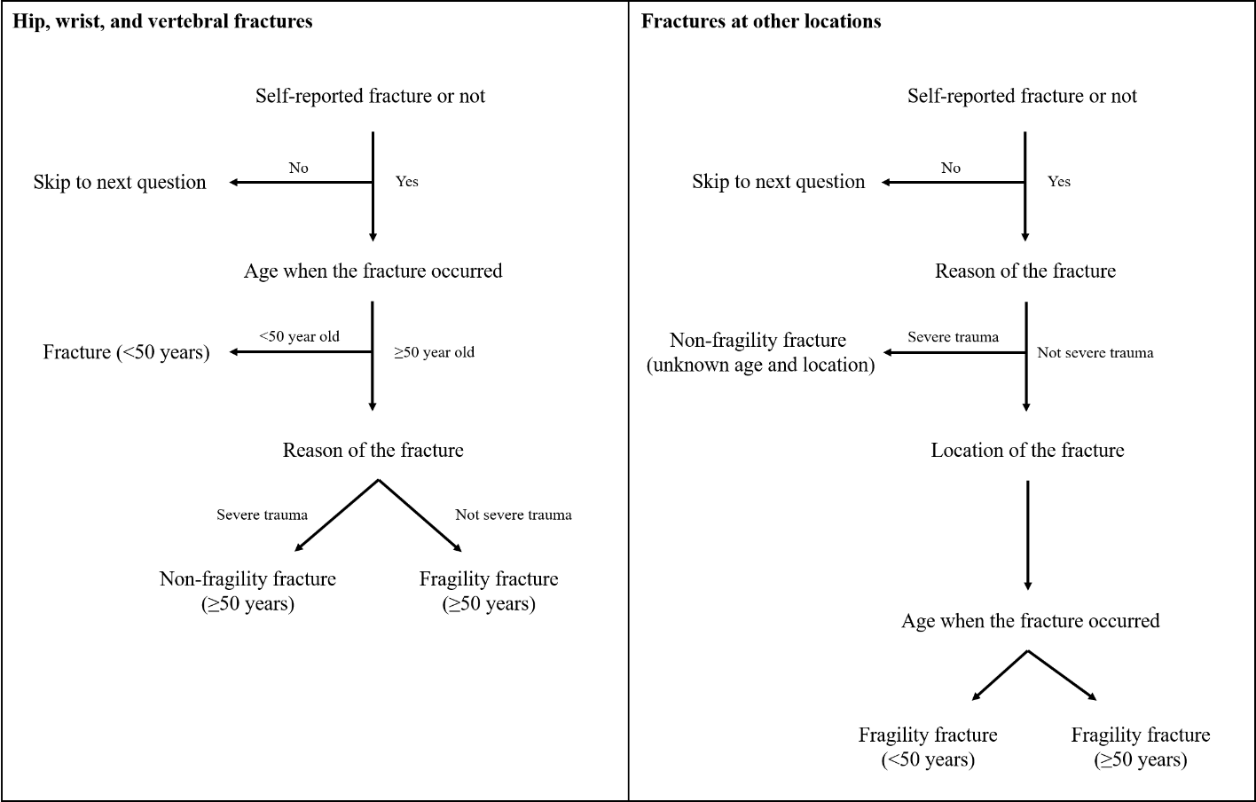


Due to the different frameworks of the fracture questionnaire, the types of fractures collected for hip, wrist, and vertebral fractures and fractures at other locations were different as follows: (1) hip, wrist, and vertebral fractures were classified into fragility fractures (≥50 years), non-fragility fractures (≥50 years), and fractures (<50 years), and (2) fractures at other locations were classified into fragility fractures (<50 years), fragility fractures (≥50 years), and non-fragility fractures with unknown age when the fracture occurred and unknown location.

## Appendix S3. Guidelines for fractures and osteoporosis published over the past 10 years

We searched the guidelines for fractures and osteoporosis in the past 10 years through the MEDLINE database via Ovid on August 18^th^ 2023. Search strategy was shown in Appendix S3.1.

**Appendix S3.1. Search strategy**

| **#** | **Searches** | **Results** |
| --- | --- | --- |
| 1 | exp Fractures, Bone/ | 209089 |
| 2 | Broken Bone$.ab,ti. | 355 |
| 3 | Bone Fracture$.ab,ti. | 12710 |
| 4 | Spiral Fracture$.ab,ti. | 393 |
| 5 | Torsion Fracture$.ab,ti. | 43 |
| 6 | 1 or 2 or 3 or 4 or 5 | 215826 |
| 7 | Osteoporosis/ | 50813 |
| 8 | osteop$.tw. | 131814 |
| 9 | bone density/ | 60925 |
| 10 | (bone adj2 densit$).tw. | 63109 |
| 11 | bmd.tw. | 34579 |
| 12 | exp "Bone and Bones"/ | 677559 |
| 13 | bone loss$.tw. | 35713 |
| 14 | 7 or 8 or 9 or 10 or 11 or 12 or 13 | 822205 |
| 15 | 6 or 14 | 928398 |
| 16 | guideline*.ti. | 93794 |
| 17 | guidance*.ti. | 21406 |
| 18 | position paper.ti. | 3077 |
| 19 | position stand.ti. | 69 |
| 20 | statement*.ti. | 21751 |
| 21 | recommendation*.ti. | 50135 |
| 22 | consensus.ti. | 32736 |
| 23 | practice parameter*.ti. | 692 |
| 24 | standards.ti. | 26379 |
| 25 | management*.ti. | 459149 |
| 26 | 16 or 17 or 18 or 19 or 20 or 21 or 22 or 23 or 24 or 25 | 668758 |
| 27 | 15 and 26 | 23018 |
| 28 | limit 27 to (humans and yr="2013 -Current") | 9027 |

**Appendix S3.2. PRISMA flow chart of study identification, screening, and selection for the guidelines for fractures and osteoporosis**


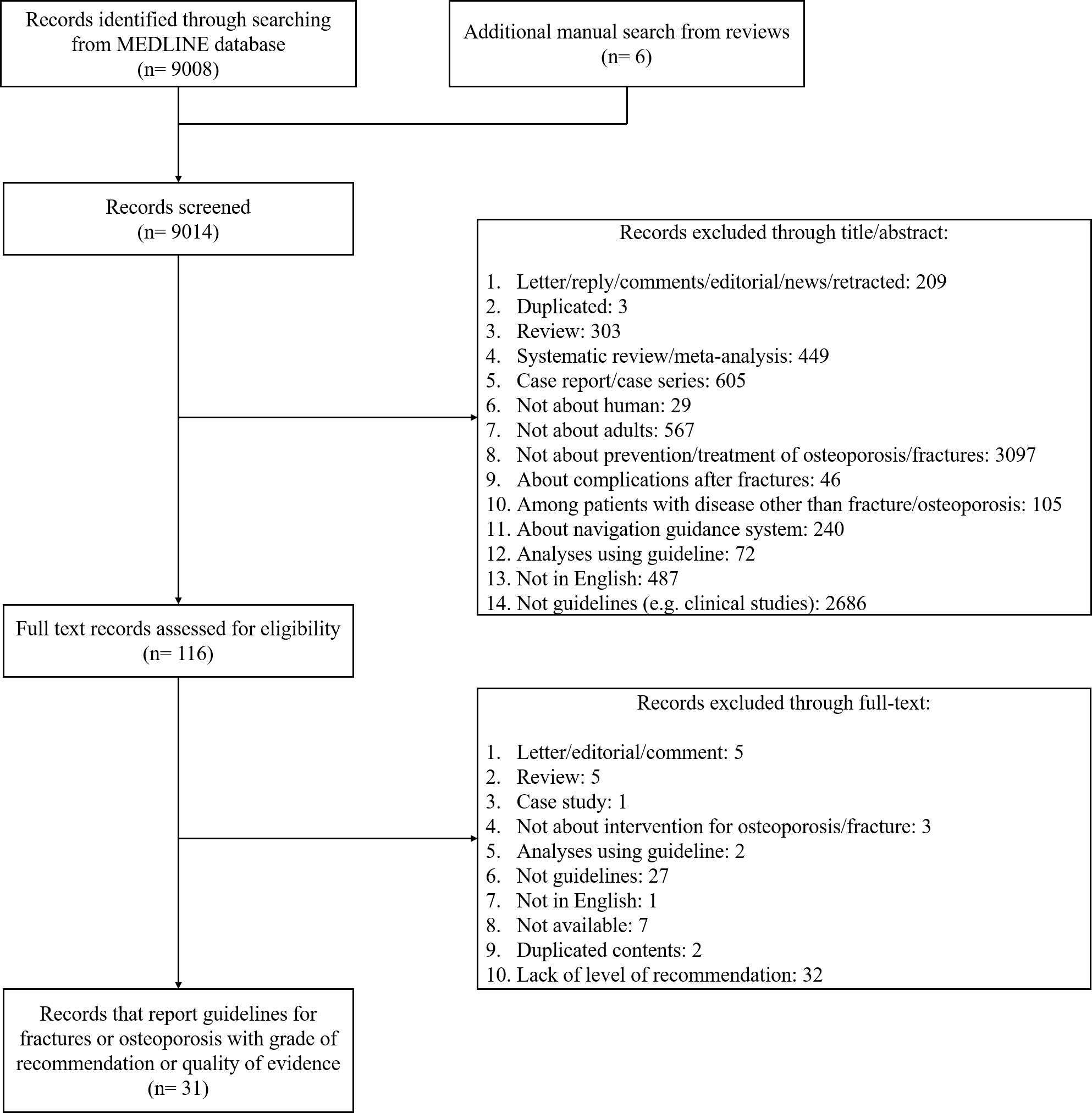


**Appendix S3.3. Published Guidelines on management of fractures published over the past 10 years**

| **No.** | **Country/Year** | **Population** | **Recommendation** |
| --- | --- | --- | --- |
| 1 | USA (2022)^2^ | Adults aged ≥50 years with hip fractures | Recommendations for management of fractures |
| 2 | USA (2022)^3^ | Patients with distal radius fractures | Recommendations for management of fractures |
| 3 | USA (2021)^4^ | Patients with distal radius fractures | Recommendations for evaluation and treatment of acute distal radius fractures |
| 4 | USA (2020)^5^ | Patients with traumatic facial fractures | Recommendations for antibiotic administration in the management of traumatic facial fractures |
| 5 | USA (2017)^6^ | Patients with rib fractures | Recommendations for operative fixation |
| 6 | USA (2015)^7^ | Elderly patients with hip fractures | Recommendations for management of fractures |
| 7 | USA (2015)^8^ | Elderly patients with hip fractures | Recommendations for management of fractures |
| 8 | Italy (2023)^9^ | NA | 1. Clinical question: How can we identify patients at imminent risk of (re)fracture?  Recommendation: Assessing the patient’s exposure to several factors associated with imminent (re)fracture risk |
|  |  | Patients at high or imminent risk of (re)fracture | 2. Clinical question: Which therapeutic strategy should be recommended in the short- and long-term treatment of patients at high or imminent risk of (re)fracture?  Recommendation: Using a sequential pharmacologic scheme from anabolic to antiresorptive drugs, mainly in patients at higher/imminent risk of fracture |
|  |  | Patients at high risk of (re)fracture | 3. Clinical question: Might it be advisable to discontinue drugs aimed at reducing the risk of adverse events in patients at high risk of (re) fracture?  Recommendation: Avoiding treatment interruption, except for serious adverse events that occur |

**Appendix S3.4. Published Guidelines on management of osteoporosis and prevention of fractures for adults ≥50 years at moderate or high risk of fractures published over the past 10 years**

| **No.** | **Country/Year** | **Population** | **Recommendation** |
| --- | --- | --- | --- |
| 1 | USA (2023)^10^ | Postmenopausal females diagnosed with primary osteoporosis | 1. ACP recommends that clinicians use bisphosphonates for initial pharmacologic treatment to reduce the risk of fractures in postmenopausal females diagnosed with primary osteoporosis |
|  |  | Males diagnosed with primary osteoporosis | 2. ACP suggests that clinicians use bisphosphonates for initial pharmacologic treatment to reduce the risk of fractures in males diagnosed with primary osteoporosis |
|  |  | Postmenopausal females diagnosed with primary osteoporosis | 3. ACP suggests that clinicians use the RANK ligand inhibitor (denosumab) as a second-line pharmacologic treatment to reduce the risk of fractures in postmenopausal females diagnosed with primary osteoporosis who have contraindications to or experience adverse effects of bisphosphonates |
|  |  | Males diagnosed with primary osteoporosis | 4. ACP suggests that clinicians use the RANK ligand inhibitor (denosumab) as a second-line pharmacologic treatment to reduce the risk of fractures in males diagnosed with primary osteoporosis who have contraindications to or experience adverse effects of bisphosphonates |
|  |  | Females with primary osteoporosis with very high risk of fracture | 5. ACP suggests that clinicians use the sclerostin inhibitor (romosozumab, moderate-certainty evidence) or recombinant PTH (teriparatide, low-certainty evidence), followed by a bisphosphonate, to reduce the risk of fractures only in females with primary osteoporosis with very high risk of fracture |
|  |  | Females over the age of 65 with low bone mass (osteopenia) | 6. ACP suggests that clinicians take an individualized approach regarding whether to start pharmacologic treatment with a bisphosphonate in females over the age of 65 with low bone mass (osteopenia) to reduce the risk of fractures |
| 2 | USA (2022)^11^ | patients with glucocorticoid-induced osteoporosis | Recommendations for preventive measures for fractures |
|  |  |  | Recommendations for follow-up and subsequent evaluation of risk |
|  |  |  | Recommendations for diagnostic procedures |
|  |  |  | Recommendations for treatment |
| 3 | USA (2022)^12^ | Premenopausal women | Exercise Recommendations for Premenopausal Women to Slow Decline of BMD of the Femoral Neck |
|  |  |  | Exercise Recommendations for Premenopausal Women to Slow Decline of BMD of the Lumbar Spine |
| 4 | USA (2020)^13^ | Postmenopausal Women | Recommendations for pharmacological Management of Osteoporosis |
| 5 | USA (2020)^14^ | Patients with postmenopausal osteoporosis | Recommendations for diagnosis and treatment of postmenopausal osteoporosis |
| 6 | USA (2017)^15^ | Men and Women with osteoporosis | Recommendations for treatment of low bone density or osteoporosis to prevent fractures |
|  |  | Osteopenic women 65 years of age or older |  |
| 7 | USA (2017)^16^ | All adults taking prednisone at a dose of ≥2.5 mg/day for ≥3 months | Optimize calcium intake (1,000–1,200 mg/day) and vitamin D intake (600–800 IU/day) and lifestyle modifications (balanced diet, maintaining weight in the recommended range, smoking cessation, regular weight-bearing or resistance training exercise, limiting alcohol intake to 1–2 alcoholic beverages/day) over no treatment or over any of these treatments alone. |
|  |  | Adults age ≥40 years at low risk of fracture | Optimize calcium and vitamin D intake and lifestyle modifications over treatment with bisphosphonates, teriparatide, denosumab, or  raloxifene.  Conditional recommendation for calcium and vitamin D over oral bisphosphonates, teriparatide, and denosumab because of low-quality  evidence on additional antifracture benefit of the alternative treatments in this low-risk group, costs, and potential harms |
|  |  |  | Strong recommendation for calcium and vitamin D over IV bisphosphonates and raloxifene because of low-quality evidence on additional  antifracture benefit in this low-risk group and their potential harms |
|  |  | Adults age ≥40 years at moderate risk of major fracture | Recommendations for prevention and treatment of glucocorticoid-induced osteoporosis |
|  |  | Adults age ≥40 years at high risk of fracture |  |
|  |  | Adults age <40 years at moderate-to-high risk of fracture | Recommendations for prevention and treatment of glucocorticoid-induced osteoporosis |
|  |  | Adults age ≥30 years receiving very high-dose GCs (initial dose of prednisone ≥30 mg/day and cumulative dose >5 gm in 1 year) | Treat with an oral bisphosphonate over calcium and vitamin D alone.  Treat with an oral bisphosphonate over IV bisphosphonates, teriparatide, or denosumab. |
|  |  | Adults with organ transplant, glomerular filtration rate ≥30 ml/minute, and no evidence of metabolic bone disease who continue treatment with GCs | Treat according to the age-related guidelines for adults without transplants (Table 2), with these additional recommendations: |
|  |  | Adults age ≥40 years continuing GC treatment who have had a fracture that occurred after ≥18 months of treatment with an oral bisphosphonate or who have had a significant loss of bone mineral density (≥10%/year) | Treat with another class of OP medication (teriparatide or denosumab; or, consider IV bisphosphonate if treatment failure is judged to be due to poor absorption or poor medication adherence) with calcium and vitamin D over calcium and vitamin D alone or over calcium and  vitamin D and continued oral bisphosphonate. |
|  |  | Adults age ≥40 years who have completed 5 years of oral bisphosphonate treatment and who continue GC treatment and are assessed to be at moderate-to-high risk of fracture | Recommendations for prevention and treatment of glucocorticoid-induced osteoporosis |
|  |  | Adults age ≥40 years taking an OP medication in addition to calcium and vitamin D who discontinue GC treatment and are assessed to be at low risk of fracture | Discontinue the OP medication but continue calcium and vitamin D over continuing the OP medication. |
|  |  | Adults age ≥40 years taking an OP medication in addition to calcium and vitamin D who discontinue GC treatment and are assessed to be at moderate-to-high risk of fracture | Recommendations for prevention and treatment of glucocorticoid-induced osteoporosis |
| 8 | USA (2016)^17^ | Patients with postmenopausal osteoporosis | The National Osteoporosis Foundation and American Society for Preventive Cardiology adopt the position that there is moderate-quality evidence (B level) that calcium with or without vitamin D intake from food or supplements has no relationship (beneficial or harmful) to the risk for cardiovascular and cerebrovascular disease, mortality, or all-cause mortality in generally healthy adults at this time. |
| 9 | USA (2016)^18^ | Patients with postmenopausal osteoporosis | Recommendations for diagnosis and treatment of postmenopausal osteoporosis |
| 10 | Argentina (2023)^19^ | postmenopausal women or men aged ≥50 years | Recommendations for prevention and treatment of osteoporosis |
| 11 | Brazil (2021)^20^ | Patients with glucocorticoid-induced osteoporosis | Recommendations for prevention and treatment of glucocorticoid-induced osteoporosis |
| 12 | France (2018)^21^ | Patients with postmenopausal osteoporosis | Recommendations for management of postmenopausal osteoporosis |
| 13 | India (2013)^22^ | Menopausal women | Recommendations for screening and diagnosis of osteoporosis, nutrition, and prevention of falls |
| 14 | India (2013)^23^ | Women with postmenopausal osteoporosis | Recommendations for diagnosis and management of osteoporosis |
| 15 | Italy (2017)^24^ | Patients with postmenopausal osteoporosis | Recommendations for prevention and treatment of osteoporosis |
| 16 | Italy (2016)^25^ | Patients with osteoporosis | Recommendations for treatment of osteoporosis |
|  |  | Patients with chronic kidney disease and organ transplantation | Recommendations for diagnosis, prevention and management of osteoporosis |
|  |  | NA | Osteoporosis may be a manifestation of several diseases. In 90% of cases, normal first tier laboratory results rule out other disorders of forms of secondary osteoporosis |
|  |  | Men with osteoporosis | Recommendations for diagnosis and management of osteoporosis |
|  |  | Men older than 50 years with at least two minor risk factors, men older than 70 years even in the absence of other risk factors for fracture |  |
|  |  | Men who experience a new spine or hip fracture |  |
|  |  | NA | It is recommended to achieve an adequate calcium intake through diet, limiting the use of calcium supplements to situations where this is not feasible, and only until the daily allowance has been achieved |
|  |  |  | In conjunction with an adequate calcium intake, vitamin D supplementation (cholecalciferol or ergocalciferol, i.e., D3 or D2) in the elderly has been shown useful even for primary prevention |
|  |  | Elderly | Recommendation for reduction of the risk of falls |
| 17 | Poland (2014)^26^ | Men aged >50 years and postmenopausal women | Recommendations for diagnosis and management of osteoporosis |
| 18 | Russia (2023)^27^ | postmenopausal women and men over 50 years | Recommendations for diagnosis of osteoporosis |
|  |  | patients with type 2 diabetes mellitus |  |
|  |  | Patients who have taken glucocorticoids for 3 months or more |  |
|  |  | Patients with previous major osteoporotic fracture |  |
|  |  | Patients with back pain |  |
|  |  | postmenopausal women and men over 50 years of age |  |
|  |  | patients with an individual 10-year probability of fracture (FRAX) in the interval between the low and high probability of fracture |  |
|  |  | postmenopausal women and men over 50 |  |
|  |  | patients before starting treatment for osteoporosis |  |
|  |  | Patients with postmenopausal osteoporosis and male osteoporosis |  |
|  |  | Patients who underwent surgical treatment for an osteoporotic fracture of the proximal femur |  |
|  |  | postmenopausal women with osteopenia and women over 65 with osteopenia |  |
|  |  | women with postmenopausal osteoporosis, men with increased risk of fracture, individuals with glucocorticoid-induced osteoporosis |  |
|  |  | patients with osteoporosis with a predominant decline in BMD in the cortical bone (femur neck, distal radius), patients with compromised renal function, patients for whom BP treatment was ineffective or failed to achieve the desired increase in BMD |  |
|  |  | postmenopausal women with a history of vertebral fracture,  men with osteoporosis, patients with glucocorticoid-induced osteoporosis |  |
|  |  | patients with severe osteoporosis, patients with two or more vertebral fractures, individuals where previous treatment was ineffective |  |
|  |  | patients receiving treatment for osteoporosis | Recommendations for osteoporosis treatment monitoring |
|  |  | Patients with osteoporosis |  |
|  |  | patients with previously established osteoporosis, follow-up patients |  |
|  |  | patients without fragility fractures | Recommendations for treatment for osteoporosis |
|  |  | Postmenopausal women and men above 50 years of age with a history of vertebral, hip, or multiple fractures |  |
|  |  | patients with osteoporosis without a history of previous osteoporotic fracture |  |
|  |  | Patients who were treated with teriparatide |  |
|  |  | Patients with two or more osteoporotic fractures |  |
|  |  | Women under 60 years with a postmenopausal duration of up to 10 years | Recommendations for menopausal hormone therapy in postmenopausal women to reduce the risk of fracture |
|  |  | patients aged 50 years and older with fragility fractures | Recommendations for secondary fracture prevention |
| 19 | Saudi Arabia (2015)^28^ | Postmenopausal women with osteoporosis | Recommendations for treatment of osteoporosis |
|  |  | Men with osteoporosis |  |
|  |  | Patients with glucocorticoid-induced osteoporosis |  |
|  |  | Postmenopausal women with low bone density |  |
|  |  | Patients with multiple fractures and low bone density |  |
|  |  | Postmenopausal women with severe osteoporosis |  |
|  |  | Men with severe osteoporosis |  |
|  |  | Postmenopausal women with glucocorticoid induced osteoporosis |  |
| 20 | Scotland (2021)^29^ | People over the age of 50 (with comorbidities) | Recommendations for risk factors of fractures |
|  |  | People with a parental history of osteoporosis, particularly those over the age of 50 |  |
|  |  | Women and men with low BMD |  |
|  |  | People who consume more than 3.5 units of alcohol per day |  |
|  |  | Adults with a low BMI (<20 kg/m^2^) |  |
|  |  | Patients with HIV |  |
|  |  | Patients taking oral glucocorticoids |  |
|  |  | patients with clinical risk factors for osteoporosis and whom anti-osteoporosis treatment is being considered | Recommendations for quantifying the risk of fracture |
|  |  | Patients with osteoporosis | Recommendations for management of osteoporosis and the prevention of fragility fractures |
| 21 | Spain (2015)^30^ | Female with postmenopausal, glucocorticoid-induced osteoporosis and male osteoporosis | Recommendations for treatment of osteoporosis |
| 22 | UK (2022)^31^ | postmenopausal woman, or men aged ≥50 years | Recommendations for fracture risk assessment and case finding |
|  |  | Patients whose clinical risk exceeds those factors able to be entered into FRAX |  |
|  |  | Patients with osteoporosis and/or a fragility fracture |  |
|  |  | NA | 1. Arithmetic adjustments to FRAX probabilities of major osteoporotic fracture (MOF: clinical spine, hip, forearm or humerus) and hip fracture (Table 1) can be used in clinical practice, to take account of additional clinical risk factors, such as glucocorticoid use, discordantly  low lumbar spine BMD, type II diabetes, and a history of falls. |
|  |  |  | 2. T-scores in men and women derived from femoral neck BMD should use normative values for BMD derived from young healthy women from NHANES III. |
|  |  |  | 3. DXA scan results should be reported within 3 weeks of the scan, by healthcare professionals with specific training in DXA interpretation, and in accordance with national and international reporting standards. |
|  |  |  | 4. The use of quantitative ultrasound is not recommended for the diagnosis of osteoporosis. |
|  |  | Men and women with high and very high fracture risk | Recommendations for intervention thresholds and strategy |
|  |  | Men and women with intermediate fracture risk |  |
|  |  | Very high-risk patients |  |
|  |  | NA | Arithmetic adjustments to FRAX probabilities of major osteoporotic fracture (MOF: clinical spine, hip, forearm or humerus) and hip fracture (Table 1) can be used in clinical practice, to take account of additional clinical risk factors, such as glucocorticoid use, discordantly  low lumbar spine BMD, type II diabetes, and a history of falls. |
|  |  |  | T-scores in men and women derived from femoral neck BMD should use normative values for BMD derived from young healthy women from NHANES III. |
|  |  |  | DXA scan results should be reported within 3 weeks of the scan, by healthcare professionals with specific training in DXA interpretation, and in accordance with national and international reporting standards. |
|  |  |  | The use of quantitative ultrasound is not recommended for the diagnosis of osteoporosis. |
|  |  |  | An initial FRAX assessment, which provides the ten year probability of a major osteoporotic fracture (MOF; clinical spine, hip, forearm or humerus) and/or hip fracture, can be used to identify patients at low, intermediate, high or very high risk of fracture. |
|  |  |  | Consider, particularly in older people, drug treatment in those with a prior and/or recent fragility fracture, with fracture risk assessment informing the choice of drug treatment. |
|  |  |  | When BMD is included in a FRAX assessment, the patient’s risk (high, very high or low) is determined by the higher of the two (MOF and hip fracture) risk assessments. |
|  |  |  | The choice of drug treatment should be informed by the level of fracture risk, additional clinical risk factors, cost-effectiveness of treatment and patient preferences. |
|  |  |  | FRAX and the link to the NOGG website should be incorporated into electronic patient health record systems. |
|  |  | Patients with osteoporosis | Recommendations for non‑pharmacological and pharmacological management of osteoporosis |
|  |  |  | Recommendations for duration and monitoring of bisphosphonate treatment |
|  |  |  | Recommendations for reassessment of fracture risk in individuals on osteoporosis drug treatment |
|  |  |  | Recommendations for rare adverse effects of long‑term bisphosphonate and denosumab treatment |
|  |  |  | Recommendations for glucocorticoid‑induced osteoporosis |
|  |  |  | Recommendations for men receiving androgen‑deprivation therapy |
|  |  |  | Recommendations for women receiving aromatase inhibitor therapy |
|  |  |  | Recommendations for management of symptomatic osteoporotic vertebral fractures |
|  |  |  | Recommendations for models of care for fracture prevention |
|  |  |  | Recommendations for training |
|  |  | NA | Recommendations for commissioners of healthcare |
| 23 | UK (2017)^32^ | Postmenopausal women and men aged 50 years or over | Recommendations for prevention and treatment of osteoporosis |

**Appendix S3.5. Published Guidelines on management of osteoporosis and prevention of fractures for adults <50 years at low risk of fractures published over the past 10 years**

| **No.** | **Country/Year** | **Population** | **Recommendation** |
| --- | --- | --- | --- |
| 1 | USA (2017)^16^ | Adults age <40 years at low risk of fracture | Optimize calcium and vitamin D intake and lifestyle modifications over treatment with bisphosphonates, teriparatide, or denosumab.  Conditional recommendation for calcium and vitamin D over oral bisphosphonates, teriparatide, and denosumab because of low-quality evidence on additional antifracture benefit of the alternative treatments, costs, and potential harms  Strong recommendation for calcium and vitamin D over IV bisphosphonates because of low-quality evidence for additional antifracture benefit in this low-risk group and potential harms |
| 2 | UK (2022)^31^ | Men and women with **low fracture risk**, without a prior fragility fracture | Men and women with low fracture risk, without a prior fragility fracture, can be reassured that their fracture risk is low and offered lifestyle advice as appropriate. |

## Appendix S4. Prevalence of hip, wrist, and vertebral fractures and fractures at other locations among non-Hispanic Asian aged ≥50 years, 2013-2020

| **Type of fractures** | | **2013-2014 (n= 2780)** | | **2017-2020 (n= 4987)** | | **Absolute differences between 2013-2014 and 2017-2020, % (95% CI)** | **Percentage changes between 2013-2014 and 2017-2020, % (95% CI)** |
| --- | --- | --- | --- | --- | --- | --- | --- |
|  |  | **No.** | **Prevalence,**  **% (95% CI)** | **No.** | **Prevalence,**  **% (95% CI)** |  |  |
| Hip fracture | | | | | |  |  |
|  | Overall hip fracture | 3 | 1.0 (0.3 to 1.8) | 6 | 1.3 (0.4 to 2.3) | 0.3 (-0.9 to 1.5) | 28.6 (-83.6 to 140.9) |
|  | Fragility hip fractures (≥50 years) | 1 | 0.3 (0.0 to 1.1) ^a^ | 1 | 0.2 (0.0 to 0.5) ^a^ | -0.2 (-1.0 to 0.6) | -50.2 (-278.5 to 178.0) |
|  | Non-fragility hip fractures (≥50 years) | 1 | 0.4 (0.0 to 1.0) ^a^ | 3 | 0.8 (0.0 to 1.7) | 0.5 (-0.6 to 1.6) | 136.8 (-169.6 to 443.3) |
|  | Hip fracture (<50 years) | 1 | 0.3 (0.0 to 1.0) ^a^ | 2 | 0.3 (0.0 to 0.9) ^a^ | 0.0 (-0.9 to 0.8) | -3.2 (-251.0 to 244.7) |
| Wrist fracture | | | | | |  |  |
|  | Overall wrist fracture | 9 | 3.5 (1.3 to 5.7) | 23 | 4.3 (2.3 to 6.2) | 0.8 (-2.2 to 3.7) | 21.8 (-61.5 to 105.0) |
|  | Fragility wrist fractures (≥50 years) | 5 | 2.2 (0.4 to 3.9) | 4 | 0.7 (0.0 to 1.4) | -1.5 (-3.4 to 0.4) | -68.0 (-154.4 to 18.5) |
|  | Non-fragility wrist fractures (≥50 years) | 1 | 0.4 (0.0 to 1.1) ^a^ | 5 | 1.2 (0.1 to 2.2) | 0.8 (-0.5 to 2.1) | 207.3 (-144.3 to 558.9) |
|  | Wrist fracture (<50 years) | 3 | 0.9 (0.0 to 2.0) ^a^ | 14 | 2.4 (1.1 to 3.7) | 1.5 (-0.2 to 3.2) | 156.1 (-23.6 to 335.9) |
| Vertebral fracture | | | | | |  |  |
|  | Overall vertebral fracture | 1 | 0.2 (0.0 to 0.4) ^a^ | 12 | 2.6 (1.3 to 3.8) | 2.4 (1.1 to 3.7) | 1555.5 (728.6 to 2382.5) |
|  | Fragility vertebral fractures (≥50 years) | 0 | 0 | 2 | 0.4 (0.0 to 1.1) ^a^ | 0.4 (-0.2 to 1.1) | NA |
|  | Non-fragility vertebral fractures (≥50 years) | 0 | 0 | 4 | 0.8 (0.2 to 1.5) | 0.8 (0.2 to 1.5) | NA |
|  | Vertebral fracture (<50 years) | 1 | 0.2 (0.0 to 0.4) ^a^ | 6 | 1.3 (0.2 to 2.5) | 1.2 (0.0 to 2.4) | 748.7 (-11.6 to 1509.1) |
| Fractures at other locations | |  |  |  |  |  |  |
|  | Overall fractures at other locations | 722 | 10.4 (5.9 to 14.8) | 1256 | 10.7 (8.5 to 12.9) | 0.3 (-4.6 to 5.3) | 3.1 (-44.5 to 50.7) |
|  | Fragility fractures at other locations | 220 | 2.0 (0.0 to 4.2) ^a^ | 464 | 5.2 (3.1 to 7.2) | 3.1 (0.2 to 6.1) | 154.8 (7.5 to 302.2) |
|  | Non-Fragility fractures at other locations | 560 | 8.0 (4.2 to 11.7) | 872 | 5.7 (3.3 to 8.2) | -2.2 (-6.7 to 2.2) | -28.1 (-83.9 to 27.7) |

Abbreviation: CI, confidence interval; NA, not available.

^a^ Lower end of 95% CI was smaller than 0.0 which was not accurate because of the small sample size, thus the value was recorded as 0.0.

## Appendix S5. Prevalence of fractures at other locations among US adults aged ≥50 years, 2005-March 2020

Fractures at other locations were fractures that occurred at locations including head/face, upper arm, lower arm, elbow, hand, fingers, shoulder, collar bone, ribs, pelvis, upper leg, lower leg, knee, ankle, heel, foot, toes, and other unspecified locations.

**Appendix S5.1. Prevalence of fractures at other locations stratified by types of fractures among US adults aged ≥50 years, 2005-March 2020**

| **Types of fractures** | | **2005-2006 (n= 2214)** | | **2007-2008 (n= 3074)** | | **2009-2010 (n= 3029)** | | **2013-2014 (n= 2780)** | | **2017-March 2020 (n= 4987)** | |
| --- | --- | --- | --- | --- | --- | --- | --- | --- | --- | --- | --- |
|  |  | **No.** | **Prevalence,**  **% (95% CI)** | **No.** | **Prevalence,**  **% (95% CI)** | **No.** | **Prevalence,**  **% (95% CI)** | **No.** | **Prevalence,**  **% (95% CI)** | **No.** | **Prevalence,**  **% (95% CI)** |
| Overall | | 640 | 31.3 (28.8 to 33.9) | 815 | 29.5 (25.2 to 33.9) | 806 | 30.4 (27.7 to 33.2) | 722 | 30.6 (28.2 to 32.9) | 1256 | 28.1 (26.0 to 30.1) |
| Non-fragility fractures | | 456 | 22.0 (19.5 to 24.4) | 573 | 20.3 (16.7 to 23.9) | 614 | 22.6 (20.2 to 25.1) | 560 | 23.1 (20.5 to 25.7) | 872 | 19.0 (17.3 to 20.7) |
| Fragility fractures | | 215 | 11.1 (9.1 to 13.0) | 292 | 11.5 (9.1 to 13.9) | 225 | 9.7 (8.0 to 11.4) | 220 | 10.4 (8.8 to 12.0) | 464 | 11.2 (10.0 to 12.4) |
|  | Head/face | 3 | 0.1 (0.0 to 0.2) | 7 | 0.3 (0.0 to 0.8) ^a^ | 10 | 0.4 (0.0 to 0.8) | 3 | 0.2 (0.0 to 0.4) | 10 | 0.3 (0.0 to 0.5) |
|  | Upper arm | 1 | 0.0 (0.0 to 0.1) | 5 | 0.2 (0.0 to 0.4) | 3 | 0.2 (0.0 to 0.4) ^a^ | 5 | 0.2 (0.0 to 0.3) | 9 | 0.2 (0.0 to 0.4) |
|  | Lower arm | 4 | 0.2 (0.0 to 0.4) | 8 | 0.2 (0.0 to 0.5) | 5 | 0.1 (0.0 to 0.3) | 4 | 0.3 (0.0 to 0.7) ^a^ | 21 | 0.3 (0.1 to 0.4) |
|  | Elbow | 8 | 0.4 (0.1 to 0.7) | 6 | 0.3 (0.0 to 0.6) | 6 | 0.3 (0.0 to 0.5) | 3 | 0.2 (0.0 to 0.5) ^a^ | 9 | 0.2 (0.0 to 0.3) |
|  | Hand | 7 | 0.3 (0.0 to 0.6) | 13 | 0.6 (0.3 to 0.9) | 18 | 0.7 (0.2 to 1.2) | 16 | 0.9 (0.4 to 1.4) | 31 | 0.8 (0.3 to 1.2) |
|  | Fingers | 47 | 2.4 (1.8 to 3.0) | 50 | 2.1 (1.0 to 3.1) | 42 | 1.5 (1.2 to 1.8) | 40 | 2.0 (1.1 to 2.8) | 71 | 1.7 (1.1 to 2.2) |
|  | Shoulder | 5 | 0.3 (0.0 to 0.6) | 11 | 0.2 (0.1 to 0.4) | 6 | 0.1 (0.0 to 0.3) | 8 | 0.4 (0.1 to 0.7) | 18 | 0.3 (0.1 to 0.4) |
|  | Collar bone | 2 | 0.1 (0.0 to 0.3) ^a^ | 3 | 0.1 (0.0 to 0.3) | 5 | 0.2 (0.0 to 0.5) | 4 | 0.2 (0.0 to 0.5) | 11 | 0.2 (0.0 to 0.4) |
|  | Ribs | 13 | 0.6 (0.4 to 0.9) | 29 | 1.2 (0.5 to 1.9) | 19 | 0.8 (0.3 to 1.3) | 18 | 1.0 (0.5 to 1.6) | 31 | 1.0 (0.5 to 1.4) |
|  | Pelvis | 3 | 0.2 (0.0 to 0.5) ^a^ | 4 | 0.1 (0.0 to 0.2) | 2 | 0.2 (0.0 to 0.4) ^a^ | 1 | 0.0 (0.0 to 0.0) | 9 | 0.2 (0.0 to 0.3) |
|  | Upper leg | 0 | 0 | 4 | 0.1 (0.0 to 0.4) ^a^ | 3 | 0.1 (0.1 to 0.2) | 2 | 0.1 (0.0 to 0.1) | 5 | 0.2 (0.0 to 0.3) |
|  | Lower leg | 15 | 0.7 (0.3 to 1.1) | 10 | 0.3 (0.1 to 0.5) | 11 | 0.4 (0.2 to 0.6) | 14 | 0.6 (0.2 to 1.1) | 15 | 0.5 (0.2 to 0.9) |
|  | Knee | 11 | 0.7 (0.3 to 1.1) | 14 | 0.4 (0.1 to 0.7) | 4 | 0.2 (0.0 to 0.3) | 7 | 0.2 (0.1 to 0.3) | 13 | 0.2 (0.0 to 0.4) |
|  | Ankle | 54 | 2.5 (1.6 to 3.5) | 68 | 2.6 (1.7 to 3.5) | 47 | 1.7 (1.3 to 2.1) | 43 | 2.0 (1.2 to 2.9) | 95 | 2.5 (1.6 to 3.4) |
|  | Heel | 2 | 0.1 (0.0 to 0.2) | 3 | 0.1 (0.0 to 0.3) | 0 | 0 | 1 | 0.1 (0.0 to 0.3) ^a^ | 5 | 0.1 (0.0 to 0.1) |
|  | Foot | 27 | 1.9 (0.9 to 2.9) | 46 | 1.7 (1.1 to 2.4) | 31 | 1.6 (0.9 to 2.3) | 35 | 2.0 (1.1 to 2.8) | 74 | 2.0 (1.2 to 2.8) |
|  | Toes | 39 | 2.0 (1.1 to 2.9) | 49 | 2.7 (1.9 to 3.4) | 40 | 2.2 (1.1 to 3.2) | 44 | 1.9 (1.1 to 2.7) | 87 | 2.2 (1.6 to 2.8) |
|  | Other unspecified | 3 | 0.1 (0.0 to 0.3) | 6 | 0.3 (0.0 to 0.7) ^a^ | 5 | 0.2 (0.0 to 0.4) | 9 | 0.4 (0.0 to 0.7) | 13 | 0.2 (0.0 to 0.4) |

**Appendix S5.1. Prevalence of fractures at other locations stratified by types of fractures among US adults aged ≥50 years, 2005-March 2020 (Continued)**

| **Types of fractures** | | **Absolute differences between 2005-2006 and 2017-2020, % (95% CI)** | **Percentage changes between 2005-2006 and 2017-2020, % (95% CI)** |
| --- | --- | --- | --- |
| Overall | | -3.3 (-6.6 to 0.0) | -10.5 (-21.0 to 0.03) |
| Non-fragility fractures | | -3.0 (-6.0 to 0.0) | -13.6 (-27.2 to -0.1) |
| Fragility fractures | | 0.1 (-2.2 to 2.4) | 1.0 (-19.8 to 21.7) |
|  | Head/face | 0.2 (-0.1 to 0.5) | 210.9 (-87.2 to 509.0) |
|  | Upper arm | 0.2 (0.0 to 0.4) | 547.7 (-101.2 to 1196.5) |
|  | Lower arm | 0.1 (-0.2 to 0.4) | 41.2 (-97.0 to 179.4) |
|  | Elbow | -0.2 (-0.5 to 0.1) | -52.6 (-142.1 to 36.9) |
|  | Hand | 0.5 (0.0 to 1.0) | 154.1 (-10.9 to 319.0) |
|  | Fingers | -0.8 (-1.6 to 0.0) | -31.1 (-64.4 to 2.1) |
|  | Shoulder | 0.0 (-0.4 to 0.3) | -10.8 (-123.5 to 101.9) |
|  | Collar bone | 0.1 (-0.2 to 0.4) | 47.6 (-157.3 to 252.5) |
|  | Ribs | 0.3 (-0.2 to 0.8) | 50.6 (-30.2 to 131.4) |
|  | Pelvis | 0.0 (-0.3 to 0.3) | -13.4 (-175.0 to 148.2) |
|  | Upper leg | 0.2 (0.0 to 0.4) | NA |
|  | Lower leg | -0.2 (-0.7 to 0.3) | -27.1 (-95.8 to 41.6) |
|  | Knee | -0.5 (-0.9 to -0.1) | -72.0 (-128.4 to -15.6) |
|  | Ankle | 0.0 (-1.3 to 1.3) | -1.0 (-52.1 to 50.1) |
|  | Heel | 0.0 (-0.2 to 0.1) | -28.7 (-196.0 to 138.5) |
|  | Foot | 0.0 (-1.2 to 1.3) | 2.3 (-64.5 to 69.1) |
|  | Toes | 0.2 (-0.9 to 1.3) | 8.5 (-45.3 to 62.2) |
|  | Other unspecified | 0.1 (-0.2 to 0.4) | 77.2 (-123.0 to 277.4) |

Abbreviation: CI, confidence interval; NA, not available.

^a^ Lower end of 95% CI was less than 0 due to insufficient sample size, thus, the lower end was recorded as 0.0.

**Appendix S5.2. Prevalence of overall fractures at other locations stratified by demographic characteristics among US adults aged ≥50 years, 2005-March 2020**

| **Characteristics** | | **2005-2006 (n= 2214)** | | **2007-2008 (n= 3074)** | | **2009-2010 (n= 3029)** | | **2013-2014 (n= 2780)** | | **2017-March 2020 (n= 4987)** | |
| --- | --- | --- | --- | --- | --- | --- | --- | --- | --- | --- | --- |
|  |  | **No.** | **Prevalence,**  **% (95% CI)** | **No.** | **Prevalence,**  **% (95% CI)** | **No.** | **Prevalence,**  **% (95% CI)** | **No.** | **Prevalence,**  **% (95% CI)** | **No.** | **Prevalence,**  **% (95% CI)** |
| Overall | | 640 | 31.3 (28.8 to 33.9) | 815 | 29.5 (25.2 to 33.9) | 806 | 30.4 (27.7 to 33.2) | 722 | 30.6 (28.2 to 32.9) | 1256 | 28.1 (26.0 to 30.1) |
| Age, years | |  |  |  |  |  |  |  |  |  |  |
|  | 50-64 | 289 | 31.5 (27.3 to 35.6) | 422 | 30.8 (25.3 to 36.3) | 394 | 30.9 (27.8 to 33.9) | 362 | 29.8 (27.1 to 32.5) | 576 | 26.1 (23.1 to 29.1) |
|  | ≥65 | 351 | 31.2 (28.5 to 33.8) | 393 | 27.6 (24.1 to 31.2) | 412 | 29.9 (26.3 to 33.4) | 360 | 31.7 (27.3 to 36.1) | 680 | 30.5 (28.1 to 32.9) |
| Sex | |  |  |  |  |  |  |  |  |  |  |
|  | Men | 335 | 32.6 (28.7 to 36.5) | 422 | 32.7 (27.6 to 37.8) | 416 | 32.2 (29.1 to 35.3) | 338 | 30.5 (26.7 to 34.2) | 624 | 27.8 (23.9 to 31.6) |
|  | Women | 305 | 30.3 (27.6 to 33.0) | 393 | 26.8 (22.0 to 31.6) | 390 | 28.9 (25.3 to 32.5) | 384 | 30.7 (28.4 to 32.9) | 632 | 28.3 (25.0 to 31.6) |
| Race and ethnicity | |  |  |  |  |  |  |  |  |  |  |
|  | Mexican American | 69 | 20.7 (15.9 to 25.6) | 87 | 19.9 (12.2 to 27.6) | 98 | 20.0 (17.3 to 22.7) | 69 | 22.4 (19.3 to 25.5) | 90 | 20.3 (16.6 to 23.9) |
|  | Non-Hispanic White | 444 | 33.3 (30.7 to 35.9) | 525 | 32.9 (27.8 to 38.0) | 518 | 33.6 (30.1 to 37.1) | 434 | 33.5 (30.8 to 36.1) | 652 | 31.7 (29.1 to 34.2) |
|  | Non-Hispanic Black | 102 | 21.7 (16.2 to 27.3) | 115 | 18.0 (14.2 to 21.9) | 106 | 19.4 (15.4 to 23.4) | 119 | 21.4 (16.9 to 25.9) | 309 | 22.2 (19.8 to 24.5) |
|  | Other races ^a^ | 21 | 35.1 (24.5 to 45.7) | 21 | 18.1 (13.0 to 23.3) | 27 | 21.2 (16.9 to 25.5) | 52 | 23.0 (16.5 to 29.5) | 116 | 18.0 (14.3 to 21.8) |
| Family income level | |  |  |  |  |  |  |  |  |  |  |
|  | PIR≤1.30 | 153 | 34.0 (29.0 to 39.0) | 200 | 29.9 (20.6 to 39.1) | 218 | 30.8 (26.3 to 35.4) | 225 | 32.7 (24.3 to 41.0) | 261 | 28.9 (22.1 to 35.7) |
|  | 1.30< PIR≤3.50 | 242 | 30.7 (27.0 to 34.4) | 288 | 27.8 (23.6 to 32.0) | 275 | 28.8 (24.2 to 33.5) | 245 | 29.4 (26.3 to 32.5) | 453 | 27.3 (23.8 to 30.8) |
|  | PIR>3.50 | 201 | 30.7 (26.4 to 35.0) | 244 | 30.8 (25.6 to 35.9) | 239 | 31.4 (26.3 to 36.5) | 202 | 31.0 (26.9 to 35.2) | 364 | 28.3 (23.7 to 32.9) |

**Appendix S5.2. Prevalence of overall fractures at other locations stratified by demographic characteristics among US adults aged ≥50 years, 2005-March 2020 (Continued)**

| **Characteristics** | | **Absolute differences between 2005-2006 and 2017-2020, % (95% CI)** | **Percentage changes between 2005-2006 and 2017-2020, % (95% CI)** |
| --- | --- | --- | --- |
|  |  |  |  |
| Overall | | -3.3 (-6.6 to 0.0) | -10.5 (-21.0 to 0.03) |
| Age, years | |  |  |
|  | 50-64 | -5.4 (-10.5 to -0.3) | -17.0 (-33.2 to -0.9) |
|  | ≥65 | -0.7 (-4.2 to 2.9) | -2.2 (-13.6 to 9.3) |
| Sex | |  |  |
|  | Men | -4.8 (-10.3 to 0.6) | -14.9 (-31.5 to 1.7) |
|  | Women | -2.0 (-6.2 to 2.3) | -6.5 (-20.5 to 7.6) |
| Race and ethnicity | |  |  |
|  | Mexican American | -0.5 (-6.5 to 5.6) | -2.3 (-31.6 to 27.0) |
|  | Non-Hispanic White | -1.6 (-5.3 to 2.0) | -4.9 (-15.9 to 6.1) |
|  | Non-Hispanic Black | 0.5 (-5.6 to 6.5) | 2.1 (-25.6 to 29.8) |
|  | Other races ^a^ | -17.1 (-28.3 to -5.9) | -48.7 (-80.7 to -16.7) |
| Family income level | |  |  |
|  | PIR≤1.30 | -5.1 (-13.5 to 3.3) | -15.0 (-39.8 to 9.8) |
|  | 1.30< PIR≤3.50 | -3.3 (-8.4 to 1.8) | -10.9 (-27.5 to 5.8) |
|  | PIR>3.50 | -2.3 (-8.6 to 4.0) | -7.7 (-28.2 to 12.9) |

Abbreviation: CI, confidence interval; PIR, the ratio of family income to poverty.

^a^ Other races including non-Hispanic Asians, multiracial, and other than Hispanic, non-Hispanic White, and non-Hispanic Black.

## Appendix S6. Trends in the prevalence of hip, wrist, and vertebral fractures stratified by demographic characteristics among US adults aged 20-49 years, 1999-2010

**Appendix S6.1. Trends in the prevalence of hip fractures stratified by demographic characteristics among US adults aged 20-49 years, 1999-2010**

| **Characteristics** | | **1999-2000** | **2001-2002** | **2003-2004** | **2005-2006** | **2007-2008** | **2009-2010** | **Absolute differences between 2009-2010 and 1999-2000, % (95% CI)** | **Percentage changes between 2009-2010 and 1999-2000, % (95% CI)** |
| --- | --- | --- | --- | --- | --- | --- | --- | --- | --- |
| Overall | | 0.4 (0.1 to 0.8) | 0.4 (0.0 to 0.7) | 0.7 (0.3 to 1.1) | 0.7 (0.4 to 1.1) | 0.7 (0.4 to 1.0) | 0.6 (0.4 to 0.9) | 0.2 (-0.2 to 0.6) | 51.3 (-48.6 to 151.2) |
| Age, years | |  |  |  |  |  |  |  |  |
|  | 20-34 | 0.7 (0.1 to 1.3) | 0.4 (0.0 to 1.0) ^a^ | 0.5 (0.1 to 1.0) | 0.4 (0.0 to 0.8) | 0.2 (0.0 to 0.5) | 0.5 (0.2 to 0.8) | -0.2 (-0.9 to 0.5) | -25.9 (-129.4 to 77.5) |
|  | 35-49 | 0.2 (0.0 to 0.4) | 0.4 (0.0 to 0.7) | 0.9 (0.2 to 1.6) | 1.0 (0.3 to 1.7) | 1.2 (0.6 to 1.8) | 0.8 (0.3 to 1.3) | 0.6 (0.1 to 1.1) | 317.4 (33.6 to 601.2) |
| Sex | |  |  |  |  |  |  |  |  |
|  | Men | 0.7 (0.1 to 1.4) | 0.2 (0.0 to 0.4) | 0.6 (0.1 to 1.2) | 0.8 (0.3 to 1.3) | 0.9 (0.4 to 1.4) | 0.6 (0.2 to 1.1) | -0.1 (-0.9 to 0.7) | -13.6 (-125.2 to 97.9) |
|  | Women | 0.1 (0.0 to 0.2) | 0.6 (0.0 to 1.3) ^a^ | 0.8 (0.2 to 1.4) | 0.7 (0.2 to 1.2) | 0.6 (0.3 to 0.9) | 0.6 (0.3 to 1.0) | 0.5 (0.2 to 0.9) | 467.8 (147.2 to 788.4) |
| Race and ethnicity | |  |  |  |  |  |  |  |  |
|  | Mexican American | 1.1 (0.7 to 1.6) | 0.3 (0.0 to 0.7) ^a^ | 0.8 (0.0 to 1.7) ^a^ | 0.6 (0.0 to 1.1) | 0.5 (0.0 to 1.0) ^a^ | 0.7 (0.0 to 1.4) | -0.4 (-1.3 to 0.4) | -39.3 (-110.6 to 32.0) |
|  | Non-Hispanic White | 0.2 (0.0 to 0.6) ^a^ | 0.2 (0.0 to 0.5) | 0.8 (0.3 to 1.4) | 0.8 (0.3 to 1.3) | 0.8 (0.4 to 1.2) | 0.6 (0.2 to 1.0) | 0.4 (-0.2 to 0.9) | 153.0 (-64.0 to 370.1) |
|  | Non-Hispanic Black | 0.9 (0.0 to 1.9) ^a^ | 0.6 (0.1 to 1.1) | 0.7 (0.2 to 1.2) | 0.7 (0.0 to 1.3) | 0.9 (0.2 to 1.6) | 1.2 (0.4 to 2.0) | 0.3 (-1.0 to 1.6) | 34.7 (-109.4 to 178.9) |
|  | Other race ^b^ | 0 | 0 | 0.1 (0.0 to 0.3) ^a^ | 0.4 (0.0 to 1.2) ^a^ | 0.8 (0.0 to 2.4) ^a^ | 0.2 (0.0 to 0.5) ^a^ | 0.2 (-0.2 to 0.5) | NA |
| Family income level | |  |  |  |  |  |  |  |  |
|  | PIR ≤1.30 | 1.1 (0.4 to 1.9) | 1.2 (0.0 to 2.5) ^a^ | 1.2 (0.3 to 2.2) | 0.8 (0.1 to 1.5) | 0.9 (0.1 to 1.7) | 1.1 (0.3 to 1.9) | -0.1 (-1.2 to 1.0) | -6.2 (-101.7 to 89.4) |
|  | 1.30< PIR ≤3.50 | 0.3 (0.0 to 0.7) ^a^ | 0.2 (0.0 to 0.5) | 0.7 (0.1 to 1.3) | 0.9 (0.2 to 1.6) | 0.6 (0.0 to 1.1) | 0.4 (0.2 to 0.7) | 0.2 (-0.4 to 0.7) | 57.0 (-127.3 to 241.3) |
|  | PIR >3.50 | 0.2 (0.0 to 0.6) ^a^ | 0.1 (0.0 to 0.3) | 0.5 (0.0 to 1.2) ^a^ | 0.5 (0.0 to 1.2) ^a^ | 0.9 (0.2 to 1.7) | 0.6 (0.0 to 1.4) ^a^ | 0.4 (-0.4 to 1.3) | 221.3 (-195.2 to 637.8) |

Abbreviation: CI, confidence interval; PIR, the ratio of family income to poverty; NA, not available.

^a^ Lower end of 95% CI was less than 0 due to insufficient sample size, thus, the lower end was recorded as 0.0.

^b^ Other races including non-Hispanic Asians, multiracial, and other than Hispanic, non-Hispanic White, and non-Hispanic Black.

**Appendix S6.2. Trends in the prevalence of wrist fractures stratified by demographic characteristics among US adults aged 20-49 years, 1999-2010**

| **Characteristics** | | **1999-2000** | **2001-2002** | **2003-2004** | **2005-2006** | **2007-2008** | **2009-2010** | **Absolute differences between 2009-2010 and 1999-2000, % (95% CI)** | **Percentage changes between 2009-2010 and 1999-2000, % (95% CI)** |
| --- | --- | --- | --- | --- | --- | --- | --- | --- | --- |
| Overall | | 10.1 (8.7 to 11.5) | 12.0 (10.4 to 13.7) | 10.5 (8.8 to 12.3) | 9.9 (8.4 to 11.5) | 10.1 (8.1 to 12.2) | 7.9 (6.5 to 9.3) | -2.2 (-4.2 to -0.3) | -21.9 (-41.3 to -2.5) |
| Age, years | |  |  |  |  |  |  |  |  |
|  | 20-34 | 10.5 (7.1 to 13.8) | 11.5 (9.3 to 13.7) | 9.5 (7.6 to 11.4) | 9.7 (7.6 to 11.8) | 8.9 (6.7 to 11.0) | 9.4 (7.1 to 11.7) | -1.1 (-5.2 to 3.0) | -10.1 (-49.3 to 29.0) |
|  | 35-49 | 9.8 (7.3 to 12.4) | 12.5 (10.8 to 14.3) | 11.5 (9.3 to 13.6) | 10.2 (8.4 to 11.9) | 11.3 (8.9 to 13.7) | 6.5 (5.2 to 7.8) | -3.3 (-6.2 to -0.4) | -33.7 (-63.1 to -4.3) |
| Sex | |  |  |  |  |  |  |  |  |
|  | Men | 12.8 (10.8 to 14.8) | 14.7 (12.2 to 17.1) | 13.2 (10.8 to 15.6) | 13.4 (10.9 to 16.0) | 13.2 (9.8 to 16.5) | 8.3 (6.1 to 10.4) | -4.5 (-7.5 to -1.6) | -35.5 (-58.5 to -12.4) |
|  | Women | 7.6 (5.6 to 9.6) | 9.5 (7.5 to 11.5) | 8.0 (6.0 to 9.9) | 6.5 (5.3 to 7.7) | 7.2 (5.6 to 8.8) | 7.6 (6.1 to 9.0) | 0.0 (-2.5 to 2.5) | -0.5 (-33.3 to 32.3) |
| Race and ethnicity | |  |  |  |  |  |  |  |  |
|  | Mexican American | 6.7 (4.8 to 8.7) | 7.6 (5.2 to 10.1) | 2.8 (1.1 to 4.5) | 5.3 (3.4 to 7.1) | 4.8 (3.6 to 6.0) | 3.7 (2.5 to 4.8) | -3.1 (-5.3 to -0.8) | -45.8 (-79.4 to -12.2) |
|  | Non-Hispanic White | 12.0 (10.1 to 13.9) | 14.3 (12.0 to 16.7) | 13.6 (11.2 to 16.0) | 12.9 (11.0 to 14.9) | 12.6 (9.9 to 15.3) | 9.9 (8.0 to 11.8) | -2.1 (-4.8 to 0.6) | -17.5 (-40.0 to 5.0) |
|  | Non-Hispanic Black | 5.7 (3.7 to 7.6) | 7.4 (4.9 to 10.0) | 4.9 (3.2 to 6.6) | 3.6 (2.4 to 4.8) | 5.6 (3.6 to 7.6) | 5.6 (3.3 to 7.9) | -0.1 (-3.1 to 3.0) | -0.9 (-54.2 to 52.4) |
|  | Other race ^a^ | 9.5 (2.0 to 17.1) | 6.9 (1.3 to 12.4) | 5.9 (2.6 to 9.2) | 2.9 (0.1 to 5.7) | 9.5 (2.3 to 16.7) | 4.9 (1.7 to 8.0) | -4.6 (-12.8 to 3.5) | -48.8 (-134.5 to 37.0) |
| Family income level | |  |  |  |  |  |  |  |  |
|  | PIR ≤1.30 | 9.6 (5.8 to 13.4) | 11.9 (9.7 to 14.1) | 8.2 (5.5 to 10.9) | 9.4 (7.4 to 11.4) | 10.3 (7.4 to 13.3) | 9.8 (8.1 to 11.5) | 0.2 (-4.0 to 4.3) | 1.6 (-41.4 to 44.5) |
|  | 1.30< PIR ≤3.50 | 10.9 (8.2 to 13.7) | 11.6 (8.4 to 14.8) | 10.0 (7.6 to 12.4) | 11.0 (7.6 to 14.4) | 9.0 (6.3 to 11.8) | 6.2 (4.6 to 7.7) | -4.8 (-7.9 to -1.6) | -43.6 (-72.4 to -14.8) |
|  | PIR >3.50 | 10.9 (8.5 to 13.4) | 13.3 (11.0 to 15.6) | 12.5 (9.2 to 15.9) | 9.5 (7.5 to 11.5) | 11.0 (7.8 to 14.1) | 8.6 (6.4 to 10.8) | -2.3 (-5.6 to 1.0) | -21.1 (-51.5 to 9.2) |

Abbreviation: CI, confidence interval; PIR, the ratio of family income to poverty.

^a^ Other races including non-Hispanic Asians, multiracial, and other than Hispanic, non-Hispanic White, and non-Hispanic Black.

**Appendix S6.3. Trends in the prevalence of vertebral fractures stratified by demographic characteristics among US adults aged 20-49 years, 1999-2010**

| **Characteristics** | | **1999-2000** | **2001-2002** | **2003-2004** | **2005-2006** | **2007-2008** | **2009-2010** | **Absolute differences between 2009-2010 and 1999-2000, % (95% CI)** | **Percentage changes between 2009-2010 and 1999-2000, % (95% CI)** |
| --- | --- | --- | --- | --- | --- | --- | --- | --- | --- |
| Overall | | 1.7 (1.0 to 2.5) | 2.1 (1.2 to 2.9) | 2.0 (1.1 to 2.8) | 2.2 (1.4 to 3.0) | 1.8 (1.3 to 2.4) | 1.3 (1.0 to 1.6) | -0.4 (-1.2 to 0.4) | -24.8 (-72.2 to 22.5) |
| Age, years | |  |  |  |  |  |  |  |  |
|  | 20-34 | 0.7 (0.1 to 1.2) | 2.1 (0.8 to 3.5) | 1.2 (0.5 to 1.8) | 1.3 (0.6 to 2.0) | 1.8 (1.0 to 2.7) | 0.8 (0.3 to 1.2) | 0.1 (-0.7 to 0.8) | 9.3 (-98.2 to 116.8) |
|  | 35-49 | 2.7 (1.2 to 4.2) | 2.0 (1.2 to 2.9) | 2.8 (1.3 to 4.3) | 3.0 (1.7 to 4.3) | 1.8 (1.1 to 2.6) | 1.8 (1.0 to 2.5) | -0.9 (-2.6 to 0.8) | -33.7 (-97.0 to 29.5) |
| Sex | |  |  |  |  |  |  |  |  |
|  | Men | 2.1 (1.0 to 3.2) | 2.6 (1.2 to 4.0) | 2.4 (0.9 to 3.8) | 2.5 (0.9 to 4.1) | 2.4 (1.5 to 3.3) | 1.2 (0.8 to 1.7) | -0.9 (-2.1 to 0.3) | -41.3 (-99.1 to 16.5) |
|  | Women | 1.3 (0.4 to 2.3) | 1.6 (0.6 to 2.6) | 1.6 (1.0 to 2.2) | 1.9 (0.8 to 3.0) | 1.3 (0.5 to 2.1) | 1.3 (0.8 to 1.9) | 0.0 (-1.1 to 1.1) | -0.3 (-83.2 to 82.6) |
| Race and ethnicity | |  |  |  |  |  |  |  |  |
|  | Mexican American | 0.9 (0.6 to 1.3) | 0.8 (0.3 to 1.3) | 0.7 (0.2 to 1.2) | 1.4 (0.6 to 2.2) | 1.4 (0.6 to 2.1) | 0.9 (0.2 to 1.7) | 0.0 (-0.9 to 0.9) | 0.2 (-90.5 to 90.9) |
|  | Non-Hispanic White | 2.0 (1.0 to 2.9) | 2.9 (1.8 to 4.0) | 2.5 (1.5 to 3.6) | 2.7 (1.6 to 3.9) | 2.2 (1.5 to 3.0) | 1.7 (1.3 to 2.1) | -0.3 (-1.4 to 0.8) | -14.3 (-69.4 to 40.8) |
|  | Non-Hispanic Black | 0.5 (0.0 to 1.0) | 0.4 (0.0 to 0.9) ^a^ | 0.8 (0.2 to 1.5) | 0.7 (0.2 to 1.1) | 0.7 (0.1 to 1.3) | 0.6 (0.0 to 1.2) ^a^ | 0.1 (-0.7 to 0.9) | 22.4 (-148.6 to 193.5) |
|  | Other race ^b^ | 1.7 (0.0 to 3.6) ^a^ | 0.1 (0.0 to 0.2) ^a^ | 2.2 (0.0 to 5.4) ^a^ | 2.8 (0.0 to 6.7) ^a^ | 1.4 (0.0 to 3.5) ^a^ | 0.3 (0.0 to 0.8) ^a^ | -1.3 (-3.3 to 0.7) | -80.5 (-201.2 to 40.2) |
| Family income level | |  |  |  |  |  |  |  |  |
|  | PIR ≤1.30 | 2.1 (0.8 to 3.5) | 2.2 (0.5 to 3.9) | 2.0 (0.6 to 3.3) | 2.2 (1.2 to 3.2) | 2.1 (0.8 to 3.4) | 1.7 (0.8 to 2.5) | -0.5 (-2.1 to 1.1) | -21.3 (-95.9 to 53.3) |
|  | 1.30< PIR ≤3.50 | 1.8 (0.9 to 2.7) | 2.7 (1.3 to 4.1) | 2.1 (1.0 to 3.2) | 2.6 (1.1 to 4.2) | 1.8 (0.8 to 2.8) | 1.1 (0.3 to 1.8) | -0.7 (-1.9 to 0.5) | -39.1 (-104.5 to 26.3) |
|  | PIR >3.50 | 1.9 (0.2 to 3.6) | 1.7 (0.6 to 2.7) | 1.8 (0.6 to 3.0) | 2.0 (1.0 to 3.0) | 1.9 (0.9 to 2.9) | 1.2 (0.4 to 2.0) | -0.6 (-2.5 to 1.2) | -33.9 (-132.9 to 65.0) |

Abbreviation: CI, confidence interval; PIR, the ratio of family income to poverty.

^a^ Lower end of 95% CI was less than 0 due to insufficient sample size, thus, the lower end was recorded as 0.0.

^b^ Other races including non-Hispanic Asians, multiracial, and other than Hispanic, non-Hispanic White, and non-Hispanic Black.

## Appendix S7. Prevalence of fractures at other locations among US adults aged 20-49 years, 2005-2010

Fractures at other locations were fractures that occurred at locations including head/face, upper arm, lower arm, elbow, hand, fingers, shoulder, collar bone, ribs, pelvis, upper leg, lower leg, knee, ankle, heel, foot, toes, and other unspecified locations.

**Appendix S7.1. Prevalence of fractures at other locations stratified by types of fractures among US adults aged 20-49 years, 2005-2010**

| **Types of fractures** | | **2005-2006 (n= 2765)** | | **2007-2008 (n= 2861)** | | **2009-2010 (n= 3189)** | | **Absolute differences between 2005-2006 and 2009-2010, % (95% CI)** | **Percentage changes between 2005-2006 and 2009-2010, % (95% CI)** |
| --- | --- | --- | --- | --- | --- | --- | --- | --- | --- |
|  |  | **No.** | **Prevalence,**  **% (95% CI)** | **No.** | **Prevalence,**  **% (95% CI)** | **No.** | **Prevalence,**  **% (95% CI)** |  |  |
| Overall | | 414 | 18.0 (15.7 to 20.4) | 449 | 17.7 (15.6 to 19.7) | 501 | 16.8 (15.1 to 18.5) | -1.2 (-4.2 to 1.7) | -6.9 (-23.1 to 9.3) |
| Non-fragility fractures | | 287 | 12.5 (10.6 to 14.3) | 332 | 13.0 (11.0 to 15.1) | 384 | 12.7 (11.2 to 14.2) | 0.2 (-2.1 to 2.6) | 1.8 (-17.1 to 20.7) |
| Fragility fractures | | 145 | 6.4 (5.2 to 7.5) | 139 | 5.5 (4.3 to 6.6) | 128 | 4.5 (3.3 to 5.6) | -1.9 (-3.6 to -0.2) | -30.0 (-56.1 to -3.8) |
|  | Head/face | 3 | 0.1 (0.0 to 0.2) | 5 | 0.1 (0.0 to 0.2) | 2 | 0.1 (0.0 to 0.3) | 0.0 (-0.2 to 0.2) | 14.5 (-198.7 to 227.7) |
|  | Upper arm | 1 | 0.0 (0.0 to 0.1) | 0 | 0 | 1 | 0.0 (0.0 to 0.0) | 0.0 (-0.1 to 0.0) | -45.8 (-221.5 to 129.9) |
|  | Lower arm | 1 | 0.1 (0.0 to 0.2) ^a^ | 0 | 0 | 5 | 0.2 (0.0 to 0.4) | 0.1 (-0.1 to 0.3) | 131.2 (-173.5 to 436.0) |
|  | Elbow | 4 | 0.2 (0.0 to 0.4) | 4 | 0.1 (0.0 to 0.3) | 2 | 0.1 (0.0 to 0.3) | -0.1 (-0.4 to 0.2) | -45.6 (-176.8 to 85.6) |
|  | Hand | 12 | 0.6 (0.2 to 1.0) | 15 | 0.6 (0.3 to 0.9) | 8 | 0.3 (0.1 to 0.4) | -0.3 (-0.8 to 0.1) | -56.5 (-126.6 to 13.6) |
|  | Fingers | 41 | 1.8 (1.1 to 2.5) | 32 | 1.1 (0.7 to 1.6) | 40 | 1.5 (0.9 to 2.1) | -0.3 (-1.2 to 0.6) | -17.6 (-66.8 to 31.6) |
|  | Shoulder | 2 | 0.0 (0.0 to 0.1) | 1 | 0.1 (0.0 to 0.2) ^a^ | 1 | 0.0 (0.0 to 0.0) | 0.0 (-0.1 to 0.0) | -63.8 (-213.0 to 85.4) |
|  | Collar bone | 0 | 0 | 1 | 0.0 (0.0 to 0.1) | 5 | 0.2 (0.0 to 0.5) | 0.2 (0.0 to 0.5) | NA |
|  | Ribs | 8 | 0.4 (0.1 to 0.6) | 8 | 0.2 (0.0 to 0.4) | 7 | 0.2 (0.0 to 0.4) | -0.1 (-0.4 to 0.2) | -35.6 (-121.9 to 50.7) |
|  | Pelvis | 1 | 0.0 (0.0 to 0.1) | 1 | 0.1 (0.0 to 0.2) ^a^ | 0 | 0 | 0.0 (-0.1 to 0.0) | -100.0 (-293.7 to 93.7) |
|  | Upper leg | 1 | 0.0 (0.0 to 0.1) | 1 | 0.1 (0.0 to 0.2) | 2 | 0.1 (0.0 to 0.2) | 0.0 (-0.1 to 0.2) | 144.2 (-306.9 to 595.3) |
|  | Lower leg | 6 | 0.3 (0.0 to 0.5) | 6 | 0.3 (0.0 to 0.5) | 5 | 0.2 (0.0 to 0.4) | -0.1 (-0.4 to 0.3) | -19.7 (-137.8 to 98.3) |
|  | Knee | 3 | 0.1 (0.0 to 0.2) | 2 | 0.0 (0.0 to 0.1) | 5 | 0.1 (0.0 to 0.2) | 0.0 (-0.1 to 0.2) | 41.1 (-148.2 to 230.5) |
|  | Ankle | 23 | 0.8 (0.4 to 1.2) | 23 | 0.8 (0.4 to 1.1) | 21 | 0.7 (0.4 to 1.0) | -0.1 (-0.6 to 0.4) | -14.7 (-72.9 to 43.5) |
|  | Heel | 2 | 0.1 (0.0 to 0.3) ^a^ | 0 | 0 | 1 | 0.0 (0.0 to 0.1) | -0.1 (-0.3 to 0.1) | -65.4 (-226.3 to 95.4) |
|  | Foot | 16 | 0.9 (0.4 to 1.3) | 12 | 0.6 (0.2 to 1.0) | 20 | 0.7 (0.2 to 1.2) | -0.2 (-0.8 to 0.5) | -18.3 (-95.3 to 58.7) |
|  | Toes | 33 | 1.5 (0.9 to 2.1) | 41 | 1.9 (1.3 to 2.5) | 22 | 0.8 (0.4 to 1.1) | -0.7 (-1.4 to 0.0) | -48.8 (-94.6 to -3.0) |
|  | Other unspecified | 3 | 0.1 (0.0 to 0.1) | 4 | 0.2 (0.0 to 0.3) | 1 | 0.0 (0.0 to 0.1) | 0.0 (-0.1 to 0.1) | -55.7 (-212.5 to 101.0) |

Abbreviation: CI, confidence interval; NA, not available.

^a^ Lower end of 95% CI was less than 0 due to insufficient sample size, thus, the lower end was recorded as 0.0.

**Appendix S7.2. Prevalence of overall fractures at other locations stratified by demographic characteristics among US adults aged 20-49 years, 2005-2010**

| **Characteristics** | | **2005-2006 (n= 2765)** | | **2007-2008 (n= 2861)** | | **2009-2010 (n= 3189)** | | **Absolute differences between 2005-2006 and 2009-2010, % (95% CI)** | **Percentage changes between 2005-2006 and 2009-2010, % (95% CI)** |
| --- | --- | --- | --- | --- | --- | --- | --- | --- | --- |
|  |  | **No.** | **Prevalence,**  **% (95% CI)** | **No.** | **Prevalence,**  **% (95% CI)** | **No.** | **Prevalence,**  **% (95% CI)** |  |  |
| Overall | | 414 | 18.0 (15.7 to 20.4) | 449 | 17.7 (15.6 to 19.7) | 501 | 16.8 (15.1 to 18.5) | -1.2 (-4.2 to 1.7) | -6.9 (-23.1 to 9.3) |
| Age, years | |  |  |  |  |  |  |  |  |
|  | 20-34 | 149 | 12.1 (10.2 to 14.0) | 144 | 11.6 (10.1 to 13.1) | 146 | 9.9 (8.6 to 11.1) | -2.2 (-4.5 to 0.048) | -18.4 (-37.3 to 0.4) |
|  | 35-49 | 265 | 23.3 (19.1 to 27.5) | 305 | 23.1 (20.1 to 26.2) | 355 | 23.3 (20.4 to 26.2) | 0.0 (-5.1 to 5.1) | 0.0 (-22.0 to 22.0) |
| Sex | |  |  |  |  |  |  |  |  |
|  | Men | 250 | 21.9 (17.6 to 26.1) | 266 | 22.0 (19.2 to 24.8) | 288 | 19.8 (17.4 to 22.2) | -2.1 (-7.0 to 2.8) | -9.5 (-31.8 to 12.8) |
|  | Women | 164 | 14.2 (12.1 to 16.4) | 183 | 13.4 (11.6 to 15.1) | 213 | 13.8 (11.3 to 16.4) | -0.4 (-3.8 to 2.9) | -2.9 (-26.3 to 20.5) |
| Race and ethnicity | |  |  |  |  |  |  |  |  |
|  | Mexican American | 68 | 11.7 (8.2 to 15.1) | 59 | 9.7 (5.9 to 13.6) | 70 | 10.8 (9.0 to 12.5) | -0.9 (-4.8 to 3.0) | -7.7 (-41.0 to 25.7) |
|  | Non-Hispanic White | 248 | 21.6 (18.6 to 24.5) | 271 | 21.4 (19.6 to 23.2) | 295 | 20.1 (18.0 to 22.1) | -1.5 (-5.1 to 2.2) | -6.9 (-23.8 to 10.0) |
|  | Non-Hispanic Black | 68 | 10.6 (7.7 to 13.5) | 75 | 11.8 (9.3 to 14.3) | 74 | 13.5 (9.4 to 17.6) | 2.9 (-2.1 to 7.9) | 27.4 (-19.9 to 74.7) |
|  | Other races ^b^ | 20 | 15.3 (9.3 to 21.3) | 18 | 13.7 (3.2 to 24.2) | 19 | 9.3 (5.3 to 13.4) | -6.0 (-13.3 to 1.2) | -39.2 (-86.5 to 8.0) |
| Family income level | |  |  |  |  |  |  |  |  |
|  | PIR≤1.30 | 101 | 17.0 (12.7 to 21.3) | 150 | 17.8 (13.2 to 22.4) | 172 | 15.6 (11.8 to 19.4) | -1.4 (-7.1 to 4.3) | -8.1 (-41.8 to 25.6) |
|  | 1.30< PIR≤3.50 | 142 | 17.2 (13.2 to 21.3) | 132 | 15.0 (12.8 to 17.2) | 157 | 16.2 (13.6 to 18.9) | -1.0 (-5.8 to 3.9) | -5.8 (-33.9 to 22.4) |
|  | PIR>3.50 | 147 | 18.3 (15.8 to 20.8) | 136 | 19.9 (17.3 to 22.4) | 133 | 18.2 (15.6 to 20.7) | -0.1 (-3.7 to 3.5) | -0.6 (-20.2 to 19.0) |

Abbreviation: CI, confidence interval; PIR, the ratio of family income to poverty.

^a^ Lower end of 95% CI was less than 0 due to insufficient sample size, thus, the lower end was recorded as 0.0.

^b^ Other races including non-Hispanic Asians, multiracial, and other than Hispanic, non-Hispanic White, and non-Hispanic Black.

## Appendix S8. Trends in the prevalence of anti-osteoporotic drug use and fragility hip, wrist, and vertebral fractures among US adults aged ≥50 years, 1999-March 2020

**
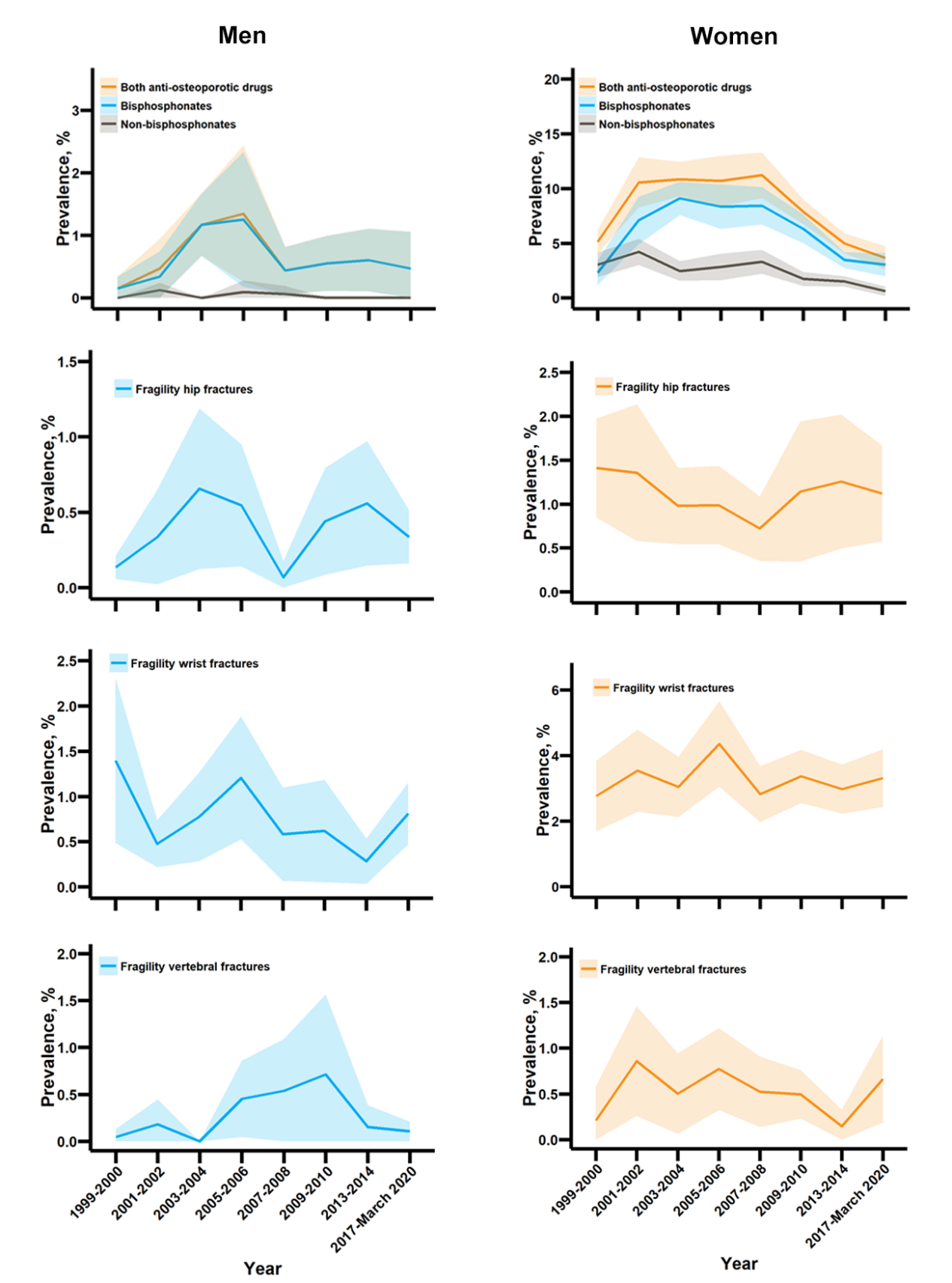
**

**Appendix S8. Trends in the prevalence of anti-osteoporotic drug use and fragility hip, wrist, and vertebral fractures among US adults aged ≥50 years, 1999-March 2020 (continued)**

| **Variables** | | **1999-2000** | **2001-2002** | **2003-2004** | **2005-2006** | **2007-2008** | **2009-2010** | **2013-2014** | **2017-March 2020** | **Relative percentage change per 2-year cycle, % (95% CI)** | ***P* value for trend** |
| --- | --- | --- | --- | --- | --- | --- | --- | --- | --- | --- | --- |
| **Men** | |  |  |  |  |  |  |  |  |  |  |
| Use of both anti-osteoporotic drugs | | 0.2 (0.0 to 0.3) | 0.5 (0.0 to 0.9) | 1.2 (0.7 to 1.7) | 1.3 (0.3 to 2.4) | 0.4 (0.1 to 0.8) | 0.6 (0.1 to 1.0) | 0.6 (0.1 to 1.1) | 0.5 (0.0 to 1.1) ^a^ | -4.9 (-24.9 to 20.3) | 0.62 |
|  | Use of bisphosphonates | 0.2 (0.0 to 0.3) | 0.3 (0.0 to 0.7) ^a^ | 1.2 (0.7 to 1.7) | 1.3 (0.2 to 2.3) | 0.4 (0.1 to 0.8) | 0.6 (0.1 to 1.0) | 0.6 (0.1 to 1.1) | 0.5 (0.0 to 1.1) ^a^ | -4.7 (-25.0 to 21.1) | 0.64 |
|  | Use of non-bisphosphonates | 0 | 0.1 (0.0 to 0.2) | 0 | 0.1 (0.0 to 0.3) ^a^ | 0.1 (0.0 to 0.2) ^a^ | 0 | 0 | 0 | NA | NA |
| Fragility hip fractures | | 0.1 (0.1 to 0.2) | 0.3 (0.0 to 0.6) | 0.7 (0.1 to 1.2) | 0.5 (0.1 to 0.9) | 0.1 (0.0 to 0.2) | 0.4 (0.1 to 0.8) | 0.6 (0.1 to 1.0) | 0.3 (0.2 to 0.5) | 6.0 (-9.0 to 23.5) | 0.39 |
| Fragility wrist fractures | | 1.4 (0.5 to 2.3) | 0.5 (0.2 to 0.7) | 0.8 (0.3 to 1.3) | 1.2 (0.5 to 1.9) | 0.6 (0.1 to 1.1) | 0.6 (0.1 to 1.2) | 0.3 (0.0 to 0.5) | 0.8 (0.5 to 1.2) | -1.7 (-12.4 to 10.4) | 0.74 |
| Fragility vertebral fractures | | 0.0 (0.0 to 0.1) | 0.2 (0.0 to 0.4) ^a^ | 0 | 0.5 (0.0 to 0.9) | 0.5 (0.0 to 1.1) | 0.7 (0.0 to 1.6) ^a^ | 0.2 (0.0 to 0.4) ^a^ | 0.1 (0.0 to 0.2) | NA | NA |
| **Women** | |  |  |  |  |  |  |  |  |  |  |
| Use of both anti-osteoporotic drugs | | 5.1 (4.1 to 6.2) | 10.0 (8.2 to 11.8) | 10.9 (9.3 to 12.4) | 10.7 (8.4 to 13.0) | 11.2 (9.1 to 13.3) | 7.9 (6.8 to 9.0) | 5.0 (4.1 to 5.9) | 3.7 (2.6 to 4.7) | -6.9 (-17.4 to 4.9) | 0.19 |
|  | Use of bisphosphonates | 2.3 (1.2 to 3.4) | 6.6 (4.9 to 8.3) | 9.1 (7.6 to 10.6) | 8.3 (6.3 to 10.4) | 8.4 (6.7 to 10.1) | 6.3 (5.0 to 7.6) | 3.5 (2.8 to 4.2) | 3.0 (2.0 to 4.1) | -10.2 (-21.4 to 2.6) | 0.10 |
|  | Use of non-bisphosphonates | 3.0 (1.9 to 4.2) | 4.1 (3.0 to 5.1) | 2.5 (1.6 to 3.4) | 2.8 (1.6 to 4.0) | 3.3 (2.2 to 4.4) | 1.7 (1.1 to 2.4) | 1.5 (1.0 to 2.0) | 0.6 (0.2 to 1.1) | -13.5 (-20.7 to -5.7) | 0.006 |
| Fragility hip fractures | | 1.4 (0.8 to 2.0) | 1.3 (0.6 to 2.1) | 1.0 (0.5 to 1.4) | 1.0 (0.5 to 1.4) | 0.8 (0.3 to 1.4) | 1.1 (0.3 to 1.9) | 1.3 (0.5 to 2.0) | 1.1 (0.6 to 1.7) | -1.4 (-6.4 to 3.8) | 0.52 |
| Fragility wrist fractures | | 2.8 (1.7 to 3.8) | 3.5 (2.3 to 4.8) | 3.1 (2.1 to 4.0) | 4.4 (3.1 to 5.7) | 2.8 (2.0 to 3.7) | 3.4 (2.6 to 4.2) | 3.0 (2.2 to 3.7) | 3.3 (2.4 to 4.2) | -0.2 (-4.7 to 4.4) | 0.90 |
| Fragility vertebral fractures | | 0.2 (0.0 to 0.6) ^a^ | 0.9 (0.3 to 1.5) | 0.6 (0.1 to 1.2) | 0.8 (0.4 to 1.3) | 0.5 (0.1 to 0.9) | 0.5 (0.2 to 0.8) | 0.1 (0.0 to 0.3) | 0.7 (0.2 to 1.1) | -4.2 (-17.3 to 11.1) | 0.51 |

Abbreviation: CI, confidence interval; NA, not available.

^a^ Lower end of 95% CI was smaller than 0.0 which was not accurate because of the small sample size, thus the value was recorded as 0.0.

## Appendix S9. Trends in the prevalence of wrist and vertebral fractures (<50 years) stratified by detailed age and sex among US adults aged ≥50 years, 1999-March 2020

**Appendix S9.1. Trends in the prevalence of wrist fractures (<50 years) stratified by detailed age and sex among US adults aged ≥50 years, 1999-March 2020**

| **Variables** | | **1999-2000** | **2001-2002** | **2003-2004** | **2005-2006** | **2007-2008** | **2009-2010** | **2013-2014** | **2017-March 2020** | **Relative percentage change per 2-year cycle, % (95% CI)** | ***P* value for trend** |
| --- | --- | --- | --- | --- | --- | --- | --- | --- | --- | --- | --- |
| **Males** | |  |  |  |  |  |  |  |  |  |  |
| 0-19 years | | 4.3 (2.2 to 6.4) | 4.9 (3.2 to 6.6) | 4.2 (1.8 to 6.6) | 6.1 (4.9 to 7.3) | 5.8 (4.3 to 7.4) | 4.4 (3.4 to 5.4) | 5.3 (3.0 to 7.5) | 6.7 (4.9 to 8.4) | 2.6 (-2.9 to 8.3) | 0.30 |
|  | 0-2 years | 0 | 0 | 0 | 0 | 0 | 0 | 0 | 0 | NA | NA |
|  | 3-5 years | 0.4 (0.0 to 0.9) ^a^ | 0.3 (0.0 to 0.7) | 0 | 0.2 (0.0 to 0.5) ^a^ | 0.3 (0.0 to 0.7) ^a^ | 0.3 (0.0 to 0.8) ^a^ | 0.1 (0.0 to 0.2) | 0.1 (0.0 to 0.3) | NA | NA |
|  | 6-11 years | 1.5 (0.5 to 2.5) | 1.9 (1.2 to 2.6) | 1.9 (0.1 to 3.6) | 2.6 (1.7 to 3.4) | 2.3 (0.9 to 3.6) | 1.6 (0.9 to 2.3) | 1.6 (0.4 to 2.9) | 2.2 (0.6 to 3.9) | 0.5 (-7.4 to 9.1) | 0.88 |
|  | 12-19 years | 2.4 (1.2 to 3.7) | 2.7 (1.4 to 4.0) | 2.3 (1.4 to 3.3) | 3.4 (2.6 to 4.1) | 3.3 (2.1 to 4.5) | 2.4 (1.7 to 3.2) | 3.5 (1.8 to 5.2) | 4.3 (3.0 to 5.6) | 5.2 (-0.2 to 10.8) | 0.06 |
| 20-49 years | | 3.2 (2.1 to 4.4) | 3.8 (2.6 to 5.1) | 4.3 (2.9 to 5.8) | 4.2 (2.8 to 5.6) | 4.4 (2.7 to 6.0) | 3.9 (2.7 to 5.0) | 4.3 (3.0 to 5.7) | 4.0 (2.9 to 5.0) | 1.0 (-1.5 to 3.5) | 0.38 |
|  | 20-29 years | 1.0 (0.1 to 1.8) | 1.7 (0.6 to 2.8) | 2.2 (0.8 to 3.6) | 2.2 (1.1 to 3.3) | 1.1 (0.4 to 1.9) | 1.3 (0.4 to 2.1) | 2.2 (1.2 to 3.2) | 1.6 (0.8 to 2.4) | 0.8 (-8.1 to 10.4) | 0.85 |
|  | 30-39 years | 0.8 (0.3 to 1.3) | 1.2 (0.4 to 2.1) | 0.7 (0.2 to 1.1) | 0.8 (0.3 to 1.4) | 1.0 (0.2 to 1.7) | 0.8 (0.0 to 1.6) | 1.1 (0.3 to 2.0) | 1.4 (0.4 to 2.3) | 4.8 (-1.6 to 11.6) | 0.12 |
|  | 40-49 years | 1.4 (0.7 to 2.2) | 0.9 (0.3 to 1.4) | 1.5 (0.7 to 2.3) | 1.1 (0.4 to 1.8) | 2.3 (1.0 to 3.6) | 1.8 (0.9 to 2.7) | 1.0 (0.3 to 1.8) | 1.0 (0.3 to 1.7) | -0.7 (-11.1 to 10.8) | 0.88 |
| **Females** | |  |  |  |  |  |  |  |  |  |  |
| 0-19 years | | 1.3 (0.5 to 2.2) | 2.3 (1.7 to 2.9) | 1.6 (0.9 to 2.3) | 3.5 (1.8 to 5.1) | 2.7 (1.7 to 3.7) | 1.6 (0.9 to 2.4) | 2.7 (1.3 to 4.0) | 4.8 (3.4 to 6.3) | 9.5 (1.1 to 18.7) | 0.03 |
|  | 0-2 years | 0.1 (0.0 to 0.4) ^a^ | 0 | 0.0 (0.0 to 0.0) | 0 | 0.3 (0.0 to 0.6) | 0 | 0.1 (0.0 to 0.3) | 0.0 (0.0 to 0.0) | NA | NA |
|  | 3-5 years | 0.1 (0.0 to 0.3) ^a^ | 0.2 (0.0 to 0.3) | 0.1 (0.0 to 0.3) ^a^ | 0.1 (0.0 to 0.2) ^a^ | 0.4 (0.0 to 0.8) ^a^ | 0.2 (0.0 to 0.4) ^a^ | 0.4 (0.0 to 1.0) ^a^ | 0.5 (0.1 to 0.9) | 16.3 (4.4 to 29.7) | 0.01 |
|  | 6-11 years | 0.8 (0.1 to 1.5) | 1.2 (0.7 to 1.7) | 0.6 (0.1 to 1.1) | 2.0 (0.9 to 3.0) | 1.2 (0.3 to 2.1) | 0.6 (0.4 to 0.9) | 1.0 (0.2 to 1.7) | 2.5 (1.5 to 3.6) | 7.8 (-6.7 to 24.6) | 0.25 |
|  | 12-19 years | 0.3 (0.0 to 0.6) ^a^ | 0.9 (0.5 to 1.3) | 0.8 (0.4 to 1.3) | 1.5 (0.5 to 2.5) | 0.8 (0.3 to 1.4) | 0.8 (0.2 to 1.5) | 1.1 (0.3 to 2.0) | 1.8 (1.2 to 2.5) | 9.4 (2.2 to 17.1) | 0.02 |
| 20-49 years | | 2.0 (0.9 to 3.2) | 2.6 (0.9 to 4.2) | 2.8 (1.9 to 3.6) | 3.3 (2.5 to 4.1) | 2.6 (1.6 to 3.7) | 3.2 (1.9 to 4.5) | 3.6 (2.2 to 5.1) | 3.6 (2.7 to 4.4) | 3.6 (0.4 to 6.9) | 0.03 |
|  | 20-29 years | 0.5 (0.2 to 0.7) | 0.4 (0.0 to 1.0) ^a^ | 0.4 (0.2 to 0.7) | 0.8 (0.1 to 1.5) | 0.6 (0.1 to 1.2) | 0.7 (0.0 to 1.4) | 0.8 (0.3 to 1.4) | 1.2 (0.2 to 2.1) | 10.6 (5.9 to 15.5) | 0.001 |
|  | 30-39 years | 0.9 (0.0 to 1.8) ^a^ | 0.7 (0.2 to 1.3) | 0.6 (0.3 to 1.0) | 1.0 (0.4 to 1.6) | 1.3 (0.5 to 2.1) | 1.0 (0.2 to 1.8) | 0.9 (0.4 to 1.5) | 0.4 (0.1 to 0.8) | -1.9 (-12.4 to 9.8) | 0.69 |
|  | 40-49 years | 0.7 (0.2 to 1.1) | 1.4 (0.1 to 2.7) | 1.7 (0.9 to 2.4) | 1.5 (0.4 to 2.6) | 0.7 (0.2 to 1.2) | 1.5 (0.8 to 2.1) | 1.9 (0.7 to 3.0) | 2.0 (1.2 to 2.7) | 6.8 (-1.4 to 15.5) | 0.09 |

Abbreviation: CI, confidence interval; NA, not available.

^a^ Lower end of 95% CI was smaller than 0.0 which was not accurate because of the small sample size, thus the value was recorded as 0.0.

**Appendix S9.2. Trends in the prevalence of vertebral fractures (<50 years) stratified by detailed age and sex among US adults aged ≥50 years, 1999-March 2020**

| **Variables** | | **1999-2000** | **2001-2002** | **2003-2004** | **2005-2006** | **2007-2008** | **2009-2010** | **2013-2014** | **2017-March 2020** | **Relative percentage change per 2-year cycle, % (95% CI)** | ***P* value for trend** |
| --- | --- | --- | --- | --- | --- | --- | --- | --- | --- | --- | --- |
| **Males** | |  |  |  |  |  |  |  |  |  |  |
| 0-19 years | | 0.3 (0.0 to 0.8) ^a^ | 0.2 (0.0 to 0.4) | 0.6 (0.0 to 1.3) | 0.1 (0.0 to 0.3) ^a^ | 1.0 (0.4 to 1.6) | 0.5 (0.0 to 0.9) | 0.1 (0.0 to 0.3) ^a^ | 0.9 (0.3 to 1.4) | 8.3 (-10.6 to 31.3) | 0.35 |
|  | 0-2 years | 0.1 (0.0 to 0.2) ^a^ | 0.1 (0.0 to 0.3) ^a^ | 0 | 0 | 0.0 (0.0 to 0.1) | 0 | 0 | 0.0 (0.0 to 0.1) | NA | NA |
|  | 3-5 years | 0 | 0 | 0 | 0 | 0 | 0 | 0.0 (0.0 to 0.1) | 0 | NA | NA |
|  | 6-11 years | 0 | 0 | 0.0 (0.0 to 0.1) | 0 | 0.0 (0.0 to 0.1) | 0.1 (0.0 to 0.3) ^a^ | 0 | 0.0 (0.0 to 0.1) | NA | NA |
|  | 12-19 years | 0.3 (0.0 to 0.7) ^a^ | 0.1 (0.0 to 0.3) ^a^ | 0.6 (0.0 to 1.2) | 0.1 (0.0 to 0.3) ^a^ | 0.9 (0.3 to 1.6) | 0.4 (0.0 to 0.8) | 0.1 (0.0 to 0.3) ^a^ | 0.8 (0.3 to 1.3) | 8.9 (-12.2 to 35.1) | 0.37 |
| 20-49 years | | 1.3 (0.5 to 2.1) | 2.0 (0.7 to 3.2) | 2.7 (1.2 to 4.2) | 3.3 (2.1 to 4.6) | 2.4 (1.3 to 3.4) | 1.6 (0.7 to 2.4) | 1.7 (0.5 to 2.9) | 2.7 (1.7 to 3.6) | 1.9 (-6.7 to 11.3) | 0.62 |
|  | 20-29 years | 0.8 (0.0 to 1.6) | 0.6 (0.1 to 1.1) | 1.6 (0.6 to 2.5) | 1.1 (0.1 to 2.0) | 0.6 (0.1 to 1.1) | 0.5 (0.0 to 1.1) | 0.7 (0.0 to 1.3) | 0.7 (0.2 to 1.2) | -4.7 (-15.3 to 7.3) | 0.36 |
|  | 30-39 years | 0.4 (0.0 to 0.9) | 0.3 (0.0 to 0.5) | 0.4 (0.0 to 0.9) ^a^ | 1.1 (0.5 to 1.6) | 0.7 (0.0 to 1.4) ^a^ | 0.6 (0.3 to 0.9) | 0.6 (0.0 to 1.2) | 1.3 (0.6 to 2.0) | 11.0 (-1.5 to 25.2) | 0.08 |
|  | 40-49 years | 0.1 (0.0 to 0.2) ^a^ | 1.1 (0.2 to 2.0) | 0.8 (0.1 to 1.5) | 1.2 (0.3 to 2.2) | 1.0 (0.4 to 1.7) | 0.4 (0.0 to 1.0) ^a^ | 0.4 (0.0 to 0.9) ^a^ | 0.7 (0.3 to 1.0) | -3.4 (-17.3 to 12.9) | 0.61 |
| **Females** | |  |  |  |  |  |  |  |  |  |  |
| 0-19 years | | 0.1 (0.0 to 0.4) ^a^ | 0.2 (0.0 to 0.6) ^a^ | 0.1 (0.0 to 0.2) ^a^ | 0.4 (0.0 to 0.8) ^a^ | 0.4 (0.2 to 0.5) | 0.6 (0.2 to 1.0) | 0.6 (0.0 to 1.2) | 0.8 (0.3 to 1.2) | 16.4 (5.0 to 29.1) | 0.01 |
|  | 0-2 years | 0.1 (0.0 to 0.3) ^a^ | 0 | 0 | 0 | 0.1 (0.0 to 0.4) ^a^ | 0 | 0.1 (0.0 to 0.2) ^a^ | 0.0 (0.0 to 0.1) | NA | NA |
|  | 3-5 years | 0 | 0 | 0 | 0 | 0.1 (0.0 to 0.2) ^a^ | 0 | 0 | 0 | NA | NA |
|  | 6-11 years | 0.0 (0.0 to 0.0) | 0 | 0.1 (0.0 to 0.2) ^a^ | 0.1 (0.0 to 0.3) ^a^ | 0.1 (0.0 to 0.2) ^a^ | 0.3 (0.0 to 0.8) ^a^ | 0.0 (0.0 to 0.0) | 0.0 (0.0 to 0.0) | NA | NA |
|  | 12-19 years | 0 | 0.2 (0.0 to 0.6) ^a^ | 0 | 0.3 (0.0 to 0.7) ^a^ | 0.1 (0.0 to 0.2) | 0.3 (0.0 to 0.7) ^a^ | 0.5 (0.0 to 1.1) | 0.7 (0.3 to 1.1) | NA | NA |
| 20-49 years | | 1.0 (0.3 to 1.7) | 0.7 (0.2 to 1.3) | 0.9 (0.2 to 1.5) | 1.1 (0.5 to 1.8) | 1.6 (0.5 to 2.6) | 1.5 (0.6 to 2.3) | 0.6 (0.1 to 1.2) | 1.5 (0.6 to 2.5) | 4.6 (-4.5 to 14.7) | 0.27 |
|  | 20-29 years | 0.3 (0.0 to 0.7) ^a^ | 0.3 (0.0 to 0.6) ^a^ | 0.1 (0.0 to 0.2) | 0.3 (0.0 to 0.7) ^a^ | 0.5 (0.0 to 1.2) ^a^ | 0.4 (0.0 to 0.8) ^a^ | 0 | 0.4 (0.0 to 0.9) ^a^ | NA | NA |
|  | 30-39 years | 0.0 (0.0 to 0.1) | 0.4 (0.0 to 0.7) | 0.4 (0.0 to 0.8) | 0.3 (0.0 to 0.7) ^a^ | 0.5 (0.0 to 0.9) | 0.6 (0.2 to 1.1) | 0.1 (0.0 to 0.4) ^a^ | 0.5 (0.0 to 1.0) ^a^ | 9.5 (-16.1 to 42.8) | 0.44 |
|  | 40-49 years | 0.6 (0.0 to 1.3) | 0.1 (0.0 to 0.2) | 0.4 (0.0 to 0.9) ^a^ | 0.5 (0.1 to 1.0) | 0.6 (0.0 to 1.2) ^a^ | 0.5 (0.0 to 1.0) ^a^ | 0.5 (0.0 to 1.1) ^a^ | 0.6 (0.2 to 1.0) | 7.3 (-5.4 to 21.7) | 0.22 |

Abbreviation: CI, confidence interval; NA, not available.

^a^ Lower end of 95% CI was smaller than 0.0 which was not accurate because of the small sample size, thus the value was recorded as 0.0.

**Appendix S9.3. Prevalence of wrist fractures that occurred before the age of 20 years among US women, 1999-March 2020**

| **Types of fractures and population** | **Prevalence of wrist fractures, % (95% CI)** | | | | | | | | |
| --- | --- | --- | --- | --- | --- | --- | --- | --- | --- |
|  | **1999-2000** | **2001-2002** | **2003-2004** | **2005-2006** | **2007-2008** | **2009-2010** | **2013-2014** | **2017-March 2020** |  |
| **Wrist fractures (0-19 years)** | | | | | | | | | |
| ≥50 years | 1.3 (0.5 to 2.2) | 2.3 (1.7 to 2.9) | 1.6 (0.9 to 2.3) | 3.5 (1.8 to 5.1) | 2.7 (1.7 to 3.7) | 1.6 (0.9 to 2.4) | 2.7 (1.3 to 4.0) | 4.8 (3.4 to 6.3) |  |
| 20-49 years | 5.8 (4.1 to 7.6) | 6.8 (5.6 to 8.1) | 5.7 (4.1 to 7.3) | 4.5 (3.4 to 5.6) | 6.1 (4.2 to 8.0) | 6.0 (4.4 to 7.6) |  |  |  |
| **Wrist fractures (0-2 years)** | | | | | | | | | |
| ≥50 years | 0.1 (0.0 to 0.4) ^a^ | 0 | 0.0 (0.0 to 0.0) | 0 | 0.3 (0.0 to 0.6) | 0 | 0.1 (0.0 to 0.3) | 0.0 (0.0 to 0.0) |  |
| 20-49 years | 0.0 (0.0 to 0.0) | 0 | 0.1 (0.0 to 0.4) ^a^ | 0.3 (0.3 to 0.4) | 0.1 (0.0 to 0.4) ^a^ | 0.1 (0.0 to 0.3) ^a^ |  |  |  |
| **Wrist fractures (3-5 years)** | | | | | | | | | |
| ≥50 years | 0.1 (0.0 to 0.3) ^a^ | 0.2 (0.0 to 0.3) | 0.1 (0.0 to 0.3) ^a^ | 0.1 (0.0 to 0.2) ^a^ | 0.4 (0.0 to 0.8) ^a^ | 0.2 (0.0 to 0.4) ^a^ | 0.4 (0.0 to 1.0) ^a^ | 0.5 (0.1 to 0.9) |  |
| 20-49 years | 0.3 (0.0 to 0.8) ^a^ | 0.3 (0.0 to 0.7) ^a^ | 1.0 (0.4 to 1.6) | 0.1 (0.0 to 0.3) ^a^ | 0.3 (0.0 to 0.6) | 0.4 (0.0 to 0.7) |  |  |  |
| **Wrist fractures (6-11 years)** | | | | | | | | | |
| ≥50 years | 0.8 (0.1 to 1.5) | 1.2 (0.7 to 1.7) | 0.6 (0.1 to 1.1) | 2.0 (0.9 to 3.0) | 1.2 (0.3 to 2.1) | 0.6 (0.4 to 0.9) | 1.0 (0.2 to 1.7) | 2.5 (1.5 to 3.6) |  |
| 20-49 years | 3.2 (2.3 to 4.1) | 2.9 (1.9 to 3.9) | 2.7 (1.4 to 4.1) | 2.5 (1.8 to 3.1) | 3.7 (1.9 to 5.6) | 3.0 (2.0 to 4.0) |  |  |  |
| **Wrist fractures (12-19 years)** | | | | | | | | | |
| ≥50 years | 0.3 (0.0 to 0.6) ^a^ | 0.9 (0.5 to 1.3) | 0.8 (0.4 to 1.3) | 1.5 (0.5 to 2.5) | 0.8 (0.3 to 1.4) | 0.8 (0.2 to 1.5) | 1.1 (0.3 to 2.0) | 1.8 (1.2 to 2.5) |  |
| 20-49 years | 2.3 (1.4 to 3.2) | 3.6 (2.5 to 4.7) | 1.8 (0.9 to 2.7) | 1.6 (0.6 to 2.5) | 2.0 (1.2 to 2.8) | 2.5 (1.4 to 3.6) |  |  |  |

Abbreviation: CI, confidence interval.

^a^ Lower end of 95% CI was smaller than 0.0 which was not accurate because of the small sample size, thus the value was recorded as 0.0.

**Reference**

1. Excellence NIfHaC. Osteoporosis: assessing the risk of fragility fracture. Accessed February 12^th^, 2023. <https://www.nice.org.uk/guidance/cg146/chapter/introduction>

2. O'Connor MI, Switzer JA. AAOS Clinical Practice Guideline Summary: Management of Hip Fractures in Older Adults. *J Am Acad Orthop Surg*. Oct 15 2022;30(20):e1291-e1296. doi:10.5435/jaaos-d-22-00125

3. Kamal RN, Shapiro LM. American Academy of Orthopaedic Surgeons/American Society for Surgery of the Hand Clinical Practice Guideline Summary Management of Distal Radius Fractures. *J Am Acad Orthop Surg*. Feb 15 2022;30(4):e480-e486. doi:10.5435/jaaos-d-21-00719

4. Shapiro LM, Kamal RN. Distal Radius Fracture Clinical Practice Guidelines-Updates and Clinical Implications. *J Hand Surg Am*. Sep 2021;46(9):807-811. doi:10.1016/j.jhsa.2021.07.014

5. Forrester JD, Wolff CJ, Choi J, Colling KP, Huston JM. Surgical Infection Society Guidelines for Antibiotic Use in Patients with Traumatic Facial Fractures. *Surg Infect (Larchmt)*. Apr 2021;22(3):274-282. doi:10.1089/sur.2020.107

6. Kasotakis G, Hasenboehler EA, Streib EW, et al. Operative fixation of rib fractures after blunt trauma: A practice management guideline from the Eastern Association for the Surgery of Trauma. *J Trauma Acute Care Surg*. Mar 2017;82(3):618-626. doi:10.1097/ta.0000000000001350

7. Roberts KC, Brox WT, Jevsevar DS, Sevarino K. Management of hip fractures in the elderly. *J Am Acad Orthop Surg*. Feb 2015;23(2):131-7. doi:10.5435/jaaos-d-14-00432

8. Brox WT, Roberts KC, Taksali S, et al. The American Academy of Orthopaedic Surgeons Evidence-Based Guideline on Management of Hip Fractures in the Elderly. *J Bone Joint Surg Am*. Jul 15 2015;97(14):1196-9. doi:10.2106/jbjs.O.00229

9. Corrao G, Biffi A, Porcu G, et al. Executive summary: Italian guidelines for diagnosis, risk stratification, and care continuity of fragility fractures 2021. *Front Endocrinol (Lausanne)*. 2023;14:1137671. doi:10.3389/fendo.2023.1137671

10. Qaseem A, Hicks LA, Etxeandia-Ikobaltzeta I, et al. Pharmacologic Treatment of Primary Osteoporosis or Low Bone Mass to Prevent Fractures in Adults: A Living Clinical Guideline From the American College of Physicians. *Ann Intern Med*. Feb 2023;176(2):224-238. doi:10.7326/m22-1034

11. Messina OD, Vidal M, Torres JAM, et al. Evidence based Latin American Guidelines of clinical practice on prevention, diagnosis, management and treatment of glucocorticoid induced osteoporosis. A 2022 update : This manuscript has been produced under the auspices of the Committee of National Societies (CNS) and the Committee of Scientific Advisors (CSA) of the International Osteoporosis Foundation (IOF). *Aging Clin Exp Res*. Nov 2022;34(11):2591-2602. doi:10.1007/s40520-022-02261-2

12. Hartley GW, Roach KE, Nithman RW, et al. Physical Therapist Management of Patients With Suspected or Confirmed Osteoporosis: A Clinical Practice Guideline From the Academy of Geriatric Physical Therapy. *J Geriatr Phys Ther*. Apr-Jun 01 2022;44(2):E106-e119. doi:10.1519/jpt.0000000000000346

13. Shoback D, Rosen CJ, Black DM, Cheung AM, Murad MH, Eastell R. Pharmacological Management of Osteoporosis in Postmenopausal Women: An Endocrine Society Guideline Update. *J Clin Endocrinol Metab*. Mar 1 2020;105(3)doi:10.1210/clinem/dgaa048

14. Camacho PM, Petak SM, Binkley N, et al. AMERICAN ASSOCIATION OF CLINICAL ENDOCRINOLOGISTS/AMERICAN COLLEGE OF ENDOCRINOLOGY CLINICAL PRACTICE GUIDELINES FOR THE DIAGNOSIS AND TREATMENT OF POSTMENOPAUSAL OSTEOPOROSIS-2020 UPDATE. *Endocr Pract*. May 2020;26(Suppl 1):1-46. doi:10.4158/gl-2020-0524suppl

15. Qaseem A, Forciea MA, McLean RM, et al. Treatment of Low Bone Density or Osteoporosis to Prevent Fractures in Men and Women: A Clinical Practice Guideline Update From the American College of Physicians. *Ann Intern Med*. Jun 6 2017;166(11):818-839. doi:10.7326/m15-1361

16. Buckley L, Guyatt G, Fink HA, et al. 2017 American College of Rheumatology Guideline for the Prevention and Treatment of Glucocorticoid-Induced Osteoporosis. *Arthritis Rheumatol*. Aug 2017;69(8):1521-1537. doi:10.1002/art.40137

17. Kopecky SL, Bauer DC, Gulati M, et al. Lack of Evidence Linking Calcium With or Without Vitamin D Supplementation to Cardiovascular Disease in Generally Healthy Adults: A Clinical Guideline From the National Osteoporosis Foundation and the American Society for Preventive Cardiology. *Ann Intern Med*. Dec 20 2016;165(12):867-868. doi:10.7326/m16-1743

18. Camacho PM, Petak SM, Binkley N, et al. AMERICAN ASSOCIATION OF CLINICAL ENDOCRINOLOGISTS AND AMERICAN COLLEGE OF ENDOCRINOLOGY CLINICAL PRACTICE GUIDELINES FOR THE DIAGNOSIS AND TREATMENT OF POSTMENOPAUSAL OSTEOPOROSIS - 2016. *Endocr Pract*. Sep 2 2016;22(Suppl 4):1-42. doi:10.4158/ep161435.Gl

19. Brance ML, Larroudé MS, Zamora NV, et al. Argentine Guidelines for the Prevention and Treatment of Glucocorticoid-Induced Osteoporosis in Postmenopausal Women and Men Aged 50 Years and Older. *J Clin Rheumatol*. Aug 1 2023;29(5):e59-e70. doi:10.1097/rhu.0000000000001951

20. Pereira RMR, Perez MO, Paula AP, et al. Guidelines for the prevention and treatment of glucocorticoid-induced osteoporosis: an update of Brazilian Society of Rheumatology (2020). *Arch Osteoporos*. Mar 1 2021;16(1):49. doi:10.1007/s11657-021-00902-z

21. Briot K, Roux C, Thomas T, et al. 2018 update of French recommendations on the management of postmenopausal osteoporosis. *Joint Bone Spine*. Oct 2018;85(5):519-530. doi:10.1016/j.jbspin.2018.02.009

22. Meeta, Digumarti L, Agarwal N, Vaze N, Shah R, Malik S. Clinical practice guidelines on menopause: An executive summary and recommendations. *J Midlife Health*. Apr 2013;4(2):77-106. doi:10.4103/0976-7800.115290

23. Meeta, Harinarayan CV, Marwah R, Sahay R, Kalra S, Babhulkar S. Clinical practice guidelines on postmenopausal osteoporosis: An executive summary and recommendations. *J Midlife Health*. Apr 2013;4(2):107-26. doi:10.4103/0976-7800.115293

24. Tarantino U, Iolascon G, Cianferotti L, et al. Clinical guidelines for the prevention and treatment of osteoporosis: summary statements and recommendations from the Italian Society for Orthopaedics and Traumatology. *J Orthop Traumatol*. Nov 2017;18(Suppl 1):3-36. doi:10.1007/s10195-017-0474-7

25. Rossini M, Adami S, Bertoldo F, et al. Guidelines for the diagnosis, prevention and management of osteoporosis. *Reumatismo*. Jun 23 2016;68(1):1-39. doi:10.4081/reumatismo.2016.870

26. Głuszko P, Lorenc RS, Karczmarewicz E, Misiorowski W, Jaworski M. Polish guidelines for the diagnosis and management of osteoporosis: a review of 2013 update. *Pol Arch Med Wewn*. 2014;124(5):255-63. doi:10.20452/pamw.2255

27. Belaya Z, Rozhinskaya L, Dedov I, et al. A summary of the Russian clinical guidelines on the diagnosis and treatment of osteoporosis. *Osteoporos Int*. Mar 2023;34(3):429-447. doi:10.1007/s00198-022-06667-6

28. Al-Saleh Y, Sulimani R, Sabico S, et al. 2015 Guidelines for Osteoporosis in Saudi Arabia: Recommendations from the Saudi Osteoporosis Society. *Ann Saudi Med*. Jan-Feb 2015;35(1):1-12. doi:10.5144/0256-4947.2015.1

29. Network SIG. Management of osteoporosis and the prevention of fragility fractures. Accessed August 30^th^, 2023. <https://www.ser.es/wp-content/uploads/2018/06/Management-of-osteoporosis-and-the-prevention.pdf>

30. González-Macías J, Del Pino-Montes J, Olmos JM, Nogués X. Clinical practice guidelines for posmenopausal, glucocorticoid-induced and male osteoporosis. Spanish Society for Research on Bone and Mineral Metabolism (3rd updated version 2014). *Rev Clin Esp (Barc)*. Dec 2015;215(9):515-26. doi:10.1016/j.rce.2015.08.003

31. Gregson CL, Armstrong DJ, Bowden J, et al. UK clinical guideline for the prevention and treatment of osteoporosis. *Arch Osteoporos*. Apr 5 2022;17(1):58. doi:10.1007/s11657-022-01061-5

32. Compston J, Cooper A, Cooper C, et al. UK clinical guideline for the prevention and treatment of osteoporosis. *Arch Osteoporos*. Dec 2017;12(1):43. doi:10.1007/s11657-017-0324-5
